# Immune modulation enables a specialist insect to benefit from antibacterial withanolides in its host plant

**DOI:** 10.1038/ncomms12530

**Published:** 2016-08-26

**Authors:** Andrea Barthel, Heiko Vogel, Yannick Pauchet, Gerhard Pauls, Grit Kunert, Astrid T. Groot, Wilhelm Boland, David G. Heckel, Hanna M. Heidel-Fischer

**Affiliations:** 1 Department of Entomology, Max Planck Institute for Chemical Ecology, Hans-Knöll-Street 8, 07745 Jena, Germany; 2 Department of Bioorganic Chemistry, Max Planck Institute for Chemical Ecology, Hans-Knöll-Street 8, 07745 Jena, Germany; 3 Department of Biochemistry, Max Planck Institute for Chemical Ecology, Hans-Knöll-Street 8, 07745 Jena, Germany; 4 Department of Biodiversity and Ecosystem Dynamics, University of Amsterdam, Sciencepark 904, 1098 XH Amsterdam, The Netherlands

## Abstract

The development of novel plant chemical defenses and counter adaptations by herbivorous insect could continually drive speciation, producing more insect specialists than generalists. One approach to test this hypothesis is to compare closely related generalist and specialist species to reveal the associated costs and benefits of these different adaptive strategies. We use the specialized moth *Heliothis subflexa*, which feeds exclusively on plants in the genus *Physalis*, and its close generalist relative *H. virescens.* Specialization on *Physalis* plants necessitates the ability to tolerate withanolides, the secondary metabolites of *Physalis* species that are known to have feeding deterrent and immune inhibiting properties for other insects. Here we find that only *H. subflexa* benefits from the antibacterial properties of withanolides, and thereby gains a higher tolerance of the pathogen *Bacillus thuringiensis*. We argue that the specialization in *H. subflexa* has been guided to a large extent by a unique role of plant chemistry on ecological immunology.

## Abstract (web summary)

Certain plants in the nightshade family contain withanolides, defensive chemicals known to be harmful to most insect herbivores. Here, Barthel *et al*. show that a moth species that is a specialist herbivore of these plants benefits from the compounds by gaining increased immunity to a pathogen.

## Graphical abstract

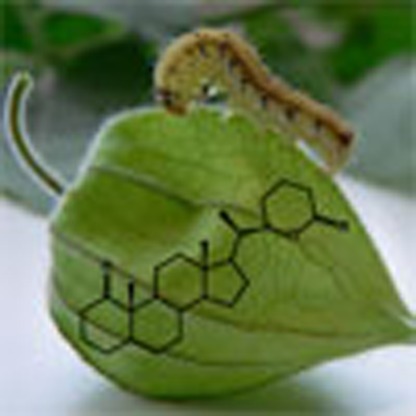

A major theme in evolutionary biology is the co-adaptation of interacting organisms. One well-known example of reciprocal adaptation is the co-evolutionary arms race between plants and their insect herbivores, as proposed in the seminal 1964 paper of Ehrlich and Raven^1^. This arms race has resulted in more specialized herbivore species consuming a few closely related species of host plants than generalized herbivore species consuming plants from several genera or families^2^,^3^. Specialized herbivores have typically evolved the ability to efficiently detoxify defensive compounds made by their hosts to deter herbivory, and to even sequester and use them to their own advantage^4^,^5^. Examples of these defensive compounds include phenolic glucosides, cardenolides and iridoid glucosides that the specialist co-opts for defence against predators, parasites and pathogens, or for interspecific communication or attracting mates^6^,^7^,^8^. There is a vast literature illustrating the great variety of methods by which specialists may benefit from host plant specialization, by utilizing plant secondary compounds to their own advantage that deter most other species.

Here we study the specialized adaptation of the noctuid moth *Heliothis subflexa* to the plant genus *Physalis*, members of this nightshade family include popular fruit and vegetables (known as cape gooseberry or simply *Physalis* fruit and tomatillo). In contrast to its close relative *Heliothis virescens,* a generalist that feeds on at least 14 different plant families but not on *Physalis*^9^,^10^, *H. subflexa* larvae feed exclusively on *Physalis* fruits^11^, and is the only *Heliothis* species to do so^12^*. H. tergemina*, the closest living relative of *H. subflexa*^13^, is a pest of tobacco in South America and has never been recorded from *Physali*s^14^, thus adaptation to *Physalis* appears uniquely in *H. subflexa* among the heliothines. *Physalis* fruits are surrounded by an inflated calyx or ‘lantern' (Supplementary Fig. 1), which shields the feeding larva from parasites and predators^15^,^16^. To utilize this enemy-free space, *H. subflexa* larvae must deal with withanolides, the major secondary plant compounds of *Physalis*^17^,^18^. Withanolides are triterpenoids with ergostan-type steroids as core structures^19^. They have been shown to be potent feeding deterrent in other insect species^20^,^21^, immunosuppressants^22^,^23^, ecdysteroid antagonists^24^ and cytotoxic agents^25^. They also have antibacterial activity^26^,^27^.

In addition to toxic secondary metabolites, herbivorous insects encounter pathogens, such as the ubiquitous entomopathogenic *Bacillus thuringiensis* (Bt). This endospore-forming bacterium produces insecticidal crystal proteins during sporulation^28^ and other toxins during vegetative growth^29^. The calyx partially shields *H. subflexa* larvae from exposure to environmental bacteria such as Bt; the generalist *H. virescens* by contrast is more exposed to the environment, feeding on the exterior of fruits of its many host plants. The different architecture of the food plants led us to hypothesize that *H. virescens* experiences stronger selection for resistance to pathogens compared with *H. subflexa*. In an earlier study, we found that when feeding on artificial diet, *H. virescens* indeed showed a stronger immune response overall than did *H. subflexa*^30^. Haemolymph phenoloxidase and lysozyme increased more in *H. virescens* after injection of pathogenic or nonpathogenic bacteria, and the haemocytes of *H. virescens* had a higher phagocytic activity and suppressed bacterial growth (including Bt) in the haemolymph more efficiently than in *H. subflexa*^30^.

With the known immunosuppressive properties of withanolides in mind, we aimed to examine specialization on *Physalis* in the context of ecological immunology. This approach combines classical studies of the immune system with an ecological perspective to evaluate the costs and benefits of defence against parasites and pathogens in the natural environment, and the manner in which natural selection shapes the immune system^31^,^32^. In tritrophic interactions between herbivorous insects, microorganisms and host plants, secondary plant compounds could either reduce intake of pathogens by reducing consumption rates of insects, or increase toxicity of pathogens by imposing additional stress on the insect's metabolism^33^. We expected that withanolides would further suppress the already relatively weak immune system of *H. subflexa*, such that the reduced pathogen defence and increased stress would have to be compensated for by the increased protection from pathogen exposure provided by the calyx. Thus, to examine the role of host plant chemistry in *H. subflexa* specialization, we tested the effects of withanolides on larval relative weight gains, survival rates and immune status of *H. subflexa* in comparison with *H. virescens.* We found that in accordance with the immunosuppressive effect seen in other insects, withanolides suppress the immune system of *H. virescens*, but unexpectedly stimulate the immune system of *H. subflexa*. Withanolides also directly inhibit the growth of some bacteria, as well as inhibiting germination of Bt spores *in vitro*. Moreover, withanolides protect *H. subflexa*, but not *H. virescens*, from the growth-suppressive effects of Bt infection. Thus, adaptation by the specialist *H. subflexa* to compounds in its host plant *Physalis* includes a novel retuning of the immune system, in which the direct antibacterial properties of withanolides are reinforced by their indirect strengthening of the immune response.

## Results

### Withanolides have a positive effect on *H. subflexa*

**Figure 1:**
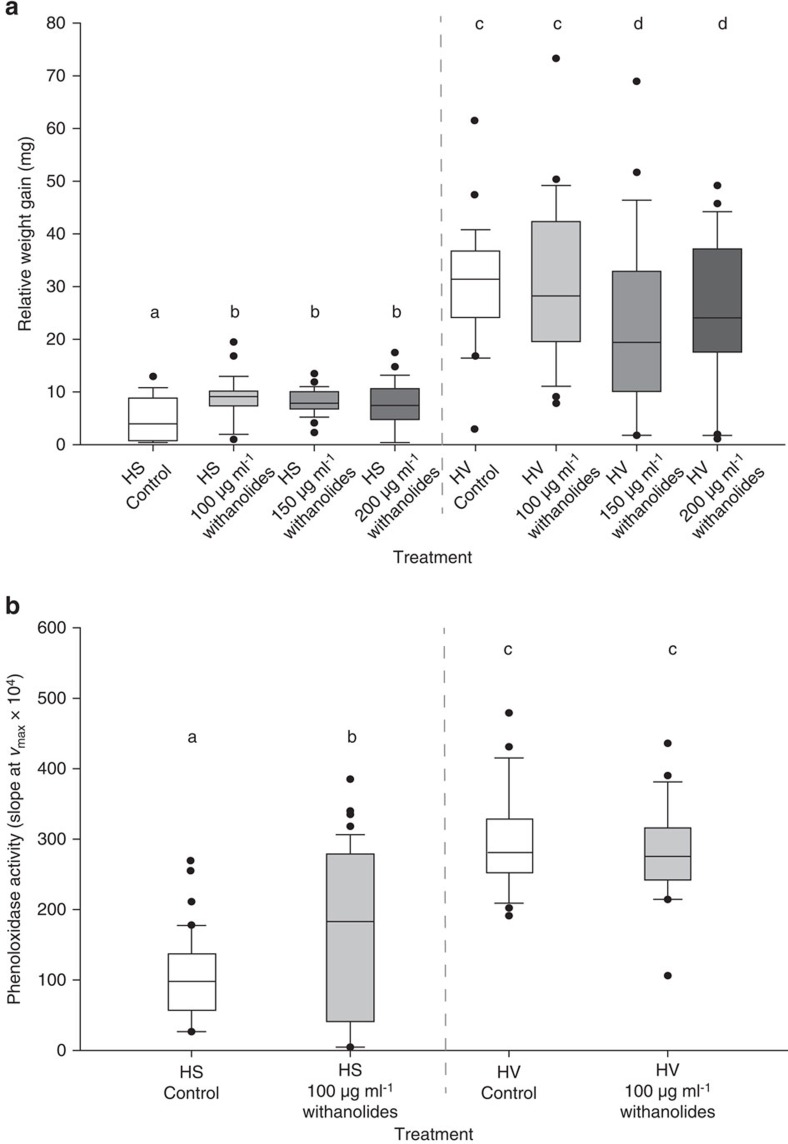
Impact of withanolides on *H. subflexa and H. virescens larvae*. (**a**) Box plot of the average relative weight gain of *H. subflexa* (HS) and *H. virescens* (HV) larvae after 7 days of exposure on diet containing 0, 100, 150 and 200 μg ml^−1^ withanolides. Increasing withanolide concentrations are indicated by grey gradient. A generalized least squares method (gls) was used to test for differences between the species (*P*=0.032; *n*=24). (**b**) Box plot of the phenoloxidase activity of HS and HV larvae after 7-day exposure to 0 μg ml^−1^ (control; white) and 100 μg ml^−1^ withanolides (grey) containing diet. The bottom and top of the box represent the 25th and 75th percentile, respectively. The horizontal line represents median value; the whiskers represent the 90th and 10th percentile and the filled circles are the extreme values. A generalized least squares method (gls) was used to test for differences between the species (*P*<0.0001, *n*=42). Letters above bars represent significant differences among treatments for all panels.

The amount of purified withanolides in the diet significantly affected the relative weight gain of *H. subflexa* larvae (Fig. 1a). Larvae reared on high withanolide concentrations, between 100 and 200 μg ml^−1^ (concentrations typically found in *Physalis* plants are 100 to 200 μg g^−1^ withanolides per fresh weight^17^), had significantly higher relative weight gain compared with individuals reared on artificial diet only (control treatment). Larvae fed on lower concentrations (<100 μg ml^−1^) of withanolides showed no significant difference in their relative weight gains compared with control treatments (Supplementary Fig. 2). The relative weight gain was significantly different between the two species (generalized least square (gls), likelihood ratio=88.903, df=1, *P*<0.001), with the generalist *H. virescens* having a higher overall weight gain than the specialist *H. subflexa* (Fig. 1a). The relative weight gain depended on the withanolide treatment (gls, likelihood ratio=7.922, df=3, *P*=0.048) and differed between the two species (gls, likelihood ratio=8.803, df=3, *P*=0.032). While the relative weight gain of *H. subflexa* larvae was significantly increased by withanolide supplementation compared with the control diet, the weight gain of larvae of *H. virescens* was similar on control diets and 100 μg ml^−1^ withanolides, and significantly lower on diets with higher concentrations of withanolides (>150 μg ml^−1^). On the basis of these results, we standardized the concentration of 100 μg ml^−1^ withanolides in our subsequent experiments, the lowest concentration that showed a significant positive effect on the relative weight gain of *H. subflexa*. When evaluating the immune status of *H. subflexa* and *H. virescens* larvae that had been previously fed on diet containing 100 μg ml^−1^ of withanolides, we found that the PO activity of *H. virescens* was significantly higher than in *H. subflexa* (Fig. 1b, gls, likelihood ratio=83.123, df=1, *P*<0.001). While there was no general influence of the withanolide treatment (gls, likelihood ratio=3.142, df=1, *P*=0.076), the PO activity significantly increased under the withanolide treatment in *H. subflexa* larvae, but not in *H. virescens* (Fig. 1b, gls, likelihood ratio=7.723, df=1, *P*=0.005).

The proportion of larvae whose haemolymph showed a measurable antibacterial activity was not influenced by the withanolide treatment (Supplementary Fig. 3a, generalized linear model (glm), with withanolides 62.7% versus without withanolides 58.5%, deviance=−0.587, df=1, *P*=0.443). In general a higher proportion of *H. virescens* than *H. subflexa* larvae showed an antibacterial activity (glm, 82.5% versus 50%, deviance=−12.861, df=1, *P*<0.001), but the withanolide treatment affected the two species in the same way (Supplementary Fig. 3a, glm, interaction: deviance=−0.176, df=1, *P*=0.675).

The strength of antibacterial response in larvae that showed measurable antibacterial activity did not depend on the withanolide treatment in both species (Supplementary Fig. 3b, two-way analysis of variance (ANOVA), F_1,71_=2.687, *P*=0.106). However, the antibacterial activity of *H. subflexa* haemolymph was significantly higher than in *H. virescens* haemolymph (two-way ANOVA, F_1,71_=86.881, *P*<0.001). The influence of the withanolide treatment on the antibacterial activity was similar for both species (two-way ANOVA, interaction: F_1,71_=0.153, *P*=0.697).

### Withanolides affect the expression of immune genes

**Figure 2:**
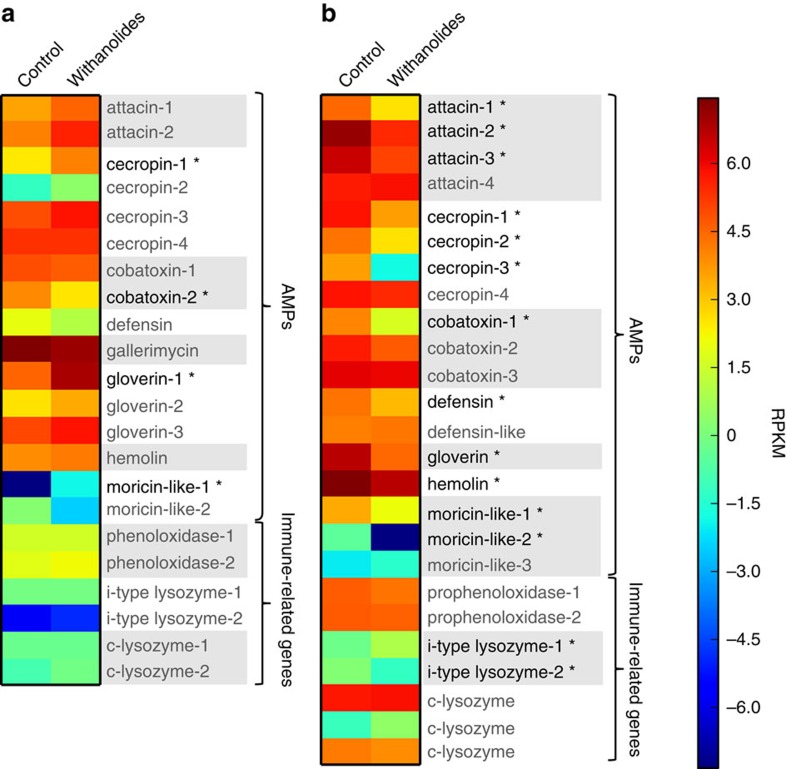
Heatmap of immune-gene expression in *H. subflexa* and *H. virescens*. Heat map showing the expression of immune genes in (**a**) *H. subflexa* and (**b**) *H. virescens* after larvae fed on control diet and diet containing 100 μg ml^−1^ withanolides. Shown are the log base 2 transformed RPKM (Reads per kilo base per million mapped reads) values. Asterisks (*) indicate significantly regulated genes (*P*<0.05, moderated *t*-test). Relative expression levels are depicted in colours from dark blue (RPKM values—6 and lower), to dark red (RPKM values 6 and higher) as shown in the colour bar to the right of the graphic.

On the transcript level, withanolides stimulate the immune system of *H. subflexa*, but inhibit the immune system of *H. virescens.* Relative expression analysis of immune-related genes measured by RNA sequencing revealed an opposite influence of withanolides on the immune system of *H. virescens* and *H. subflexa* (Fig. 2). The majority of identified immune genes in *H. virescens* larvae were downregulated or unaffected by the addition of withanolides (100 μg ml^−1^) to the diet. In total 19 of 25 genes were downregulated in larvae that had been fed on withanolide diet; 13 of those were significantly downregulated (*t*-test, df=4, *P*<0.05, see Supplementary Data 1 for *T* values). Six of the 25 genes were upregulated in larvae that had been fed on withanolide diet; with one of them being significantly upregulated (Fig. 2; Supplementary Data 1). The identified immune genes of *H. subflexa* were mostly upregulated or unaffected in larvae that had been fed on withanolide diet. Fifteen of the 22 genes were upregulated in larvae that had been fed on withanolide diet and three of these had a significant relative transcript level change (*t*-test, df=4, *P*<0.05, see Supplementary Data 1 for *T* values). Seven of the 22 genes were downregulated after withanolide treatment; with one having a significant transcript level change (*t*-test, df=4, *P*<0.05, see Supplementary Data 1 for *T* values). See Supplementary Data 1 for relative fold-change values and test statistics.

### Withanolides possess antibacterial activity

Crude *P. peruviana* leaf homogenates displayed antibacterial activity against vegetative cells of Bt, *Bacillus subtilis* and *Lactobacillus casei;* in contrast, the homogenates were inactive against *Escherichia coli*, *Serratia entomophila* and *Pseudomonas putida* (Supplementary Fig. 5; Supplementary Tables 1, 2). With the growth inhibition assays we could not differentiate between bacteriostatic and bactericidal activity, therefore we use the term antibacterial activity. We confirmed that the strong antibacterial activity of crude leaf homogenates is more pronounced in *Physalis* compared with the other plants we tested. Homogenates of leaves of other solanaceous plants such as potato, and brassicaceous plants such as rapeseed that we tested do not possess such antibacterial activity (Supplementary Fig. 5). Crude homogenates of *Physalis* leaves showed significantly higher antibacterial activity than do crude homogenates of *Physalis* calyx, rapeseed leaves and potato leaves (ANOVA, bacteria × treatment F ratio=36, df=25, *P*<0.0001; Supplementary Tables 1, 2).

**Figure 3:**
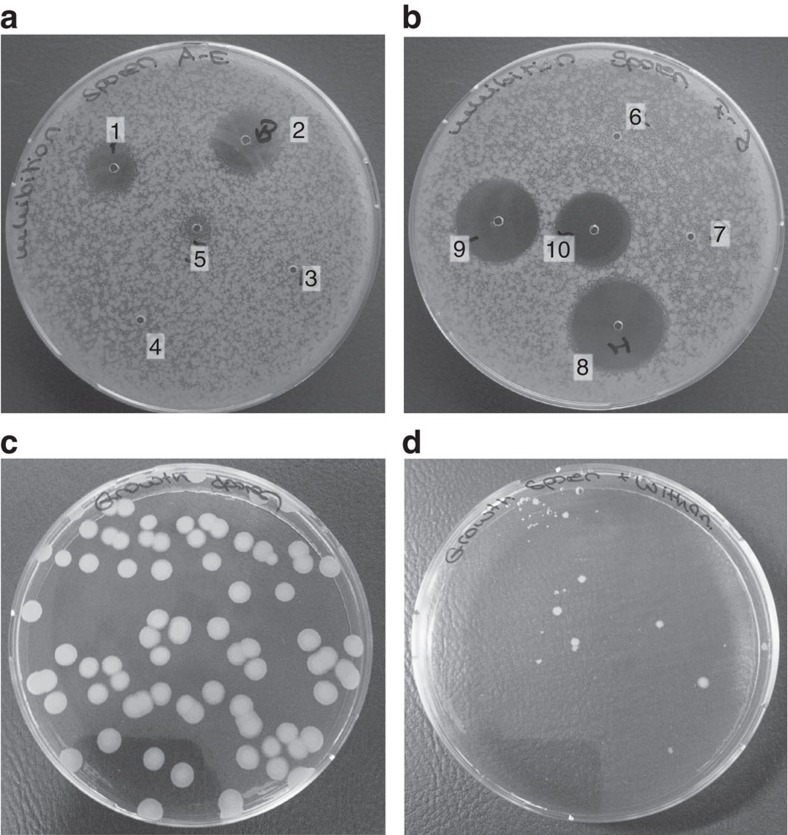
Antibacterial activity of withanolides against Bt spores. (**a**) Inhibition zone assay showing the antibacterial activity of 20 μg (10 μg μl^−1^) purified withanolides (1), 40 μg (20 μg μl^−1^) purified withanolides (2), 20 μg (10 μg μl^−1^) less purified methanolic leaf extract (3), 40 μg (20 μg μl^−1^) less purified methanolic leaf extract (4) and crude *P. peruviana* leaf homogenate (5) (**b**) Inhibition zone assay showing the antibacterial activity of 40% methanol (6), DMSO (7), 2 μg gentamycin (1 μg μl^−1^) (8), 1 μg gentamycin (0.5 μg μl^−1^) (9), 0.5 μg gentamycin (0.25 μg μl^−1^) and (10) against Bt spores. The activity of 10 μg μl^−1^ withanolides is equivalent to 0.048 μg μl^−1^ of gentamycin (see Supplementary Table 7). (**c**) Growth inhibition assay control treatment. (**d**) Growth inhibition assay showing that 100 μg ml^−1^ withanolides were able to inhibit the germination of Bt spores compared with control treatment without added withanolides. A *t*-test was used to for the comparison between control and treatment (*P*=0.038, *n*=3, see Supplementary Table 4).

To test whether the observed antibacterial activity can be related to withanolides or additional compounds found in crude *P. peruviana* leaf homogenates, we investigated the inhibitory potential of purified withanolides (methanolic leaf extracts; methanol washes passed through PS/DVB SPE cartridges) against Bt spores. Bt spores were found to be susceptible to 20 μg (10 μg μl^−1^) and 40 μg (20 μg μl^−1^) of purified withanolides from *P. peruviana* (Figs 3a). Crude *P. peruviana* leaf homogenates displayed less inhibitory activity than purified withanolides (Fig. 3a; compare 5 and 1, 2). The observed antibacterial activities of purified withanolides were significant compared with crude *P. peruviana* leaf homogenates (Overall *t*-test: F ratio 203.4, df=8, *P*<0.0001; Supplementary Fig. 6, Supplementary Table 3). However, less purified methanolic leaf extracts from *P. peruviana* leaves containing withanolides and other metabolites, for example, flavonoids (without SPE purification) did not show any antibacterial activity (Figs 3a). As a positive control, Bt spores were inhibited by 1–0.25 μg μl^−1^ gentamycin (Fig. 3b, 8–10). As a negative control, methanol (used as a solvent in leaf extracts) and dimethylsulfoxide did not show any antibacterial activity (Fig. 3b, 6 and 7).

When reproducing the conditions on *Physalis* plants (100 μg ml^−1^ withanolides inoculated in agar), we found that withanolides significantly inhibit Bt spore germination (compare Fig. 3c,d; Supplementary Fig. 7, Supplementary Table 4; *t*-test, df=1, *P*<0.038).

### Negative impact of Bt spores on H. *subflexa* larvae

**Figure 4:**
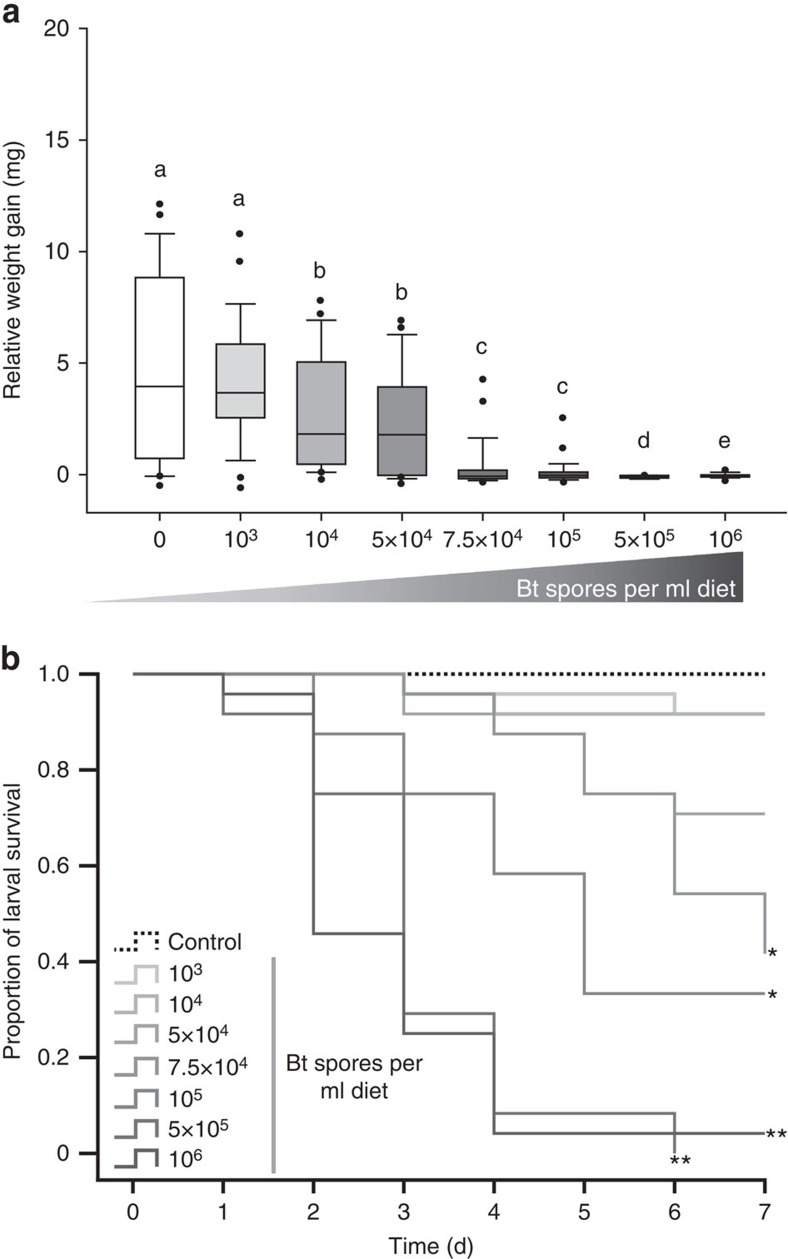
Impact of Bt spores on *H. subflexa* larvae. (**a**) Average relative weight gain of *H. subflexa* larvae after 7 days of exposure to 0–10^6^ CFU per ml Bt spores applied to artificial diet. Increasing Bt spore concentrations are indicated by a grey gradient. The bottom and top of the box represent the 25th and 75th percentile, respectively. The horizontal line represents median value. The whiskers represent the 90th and 10th percentile with and the filled circles are the extreme values. (**b**) Kaplan–Meier survival plot of larvae fed on 0–10^6^ CFU per ml Bt spores (*n*=24). Increasing Bt spore concentrations are indicated by a grey gradient. Significant differences tested by a Cox regression survival analysis are indicated by **P<0.01; *P<0.05. See Supplementary Table 5 for significance values.

Analysis of the relative weight gains of *H. subflexa* larvae fed on diet containing Bt spores ranging from 0 to 10^6^ CFU per ml (CFU, colony-forming units) showed that the weight gains were significantly negatively affected by concentrations ≥10^4^ CFU per ml Bt spores (Fig. 4a). Increasing Bt spore concentrations to 10^6^ CFU per ml caused larval relative weight gains to decrease in a dose-dependent manner. At the highest concentrations (5 × 10^5^ and 10^6^ CFU per ml), larval relative weight gains turned negative: larvae suffered very high weight loss and mortality. Thus the weight gain of *H. subflexa* depends on the Bt treatment (gls, likelihood ratio=75.874, df=6, *P*<0.001).

Analysis of the larval mortality in response to Bt spores showed that concentrations ≥7.5 × 10^5^ CFU per ml caused significantly more infected larvae to die than in control treatments (Fig. 4b; see Supplementary Table 5 for statistical significance). Larvae fed on diet containing the highest concentrations of 5 × 10^5^ CFU per ml and 10^6^ CFU per ml Bt spores showed the highest mortality rates with no or only one surviving larvae after 7 days of treatment. Equal larval mortality levels in response to 5 × 10^5^ CFU per ml were found in *H. virescens* and *H. subflexa* in earlier experiments^30^, therefore we used the lethal dose of 5 × 10^5^ CFU per ml of Bt spores for all further experiments in both species.

### Withanolides increase survival of Bt infected *H. subflexa*

**Figure 5:**
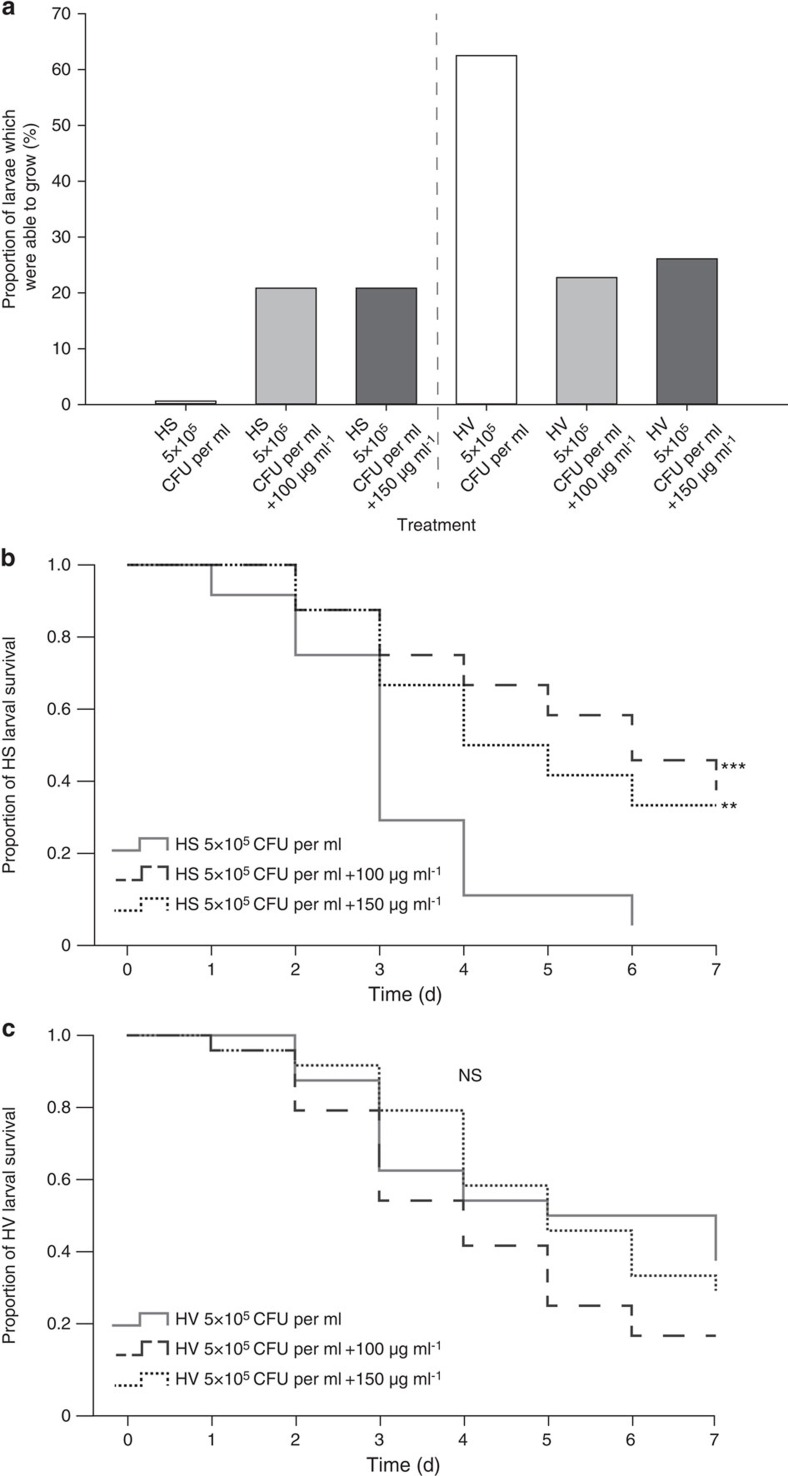
Impact of withanolides on infected *H. subflexa* and *H. virescens* larvae. (**a**) Proportion of HS and HV larvae which were able to grow on diet containing 5 × 10^5^ CFU per ml Bt spores with or without withanolide treatment. Increasing withanolide concentrations are indicated by a grey gradient. A generalized least squares method (glm) was used to test for differences between the species (*P*<0.0001). (**b**) Kaplan–Meier survival plot of HS larvae fed on (i) 5 × 10^5^ CFU per ml Bt spores, (ii) 5 × 10^5^ CFU per ml Bt spores and 100 μg ml^−1^ withanolides and (iii) 5 × 10^5^ CFU per ml Bt spores and 150 μg ml^−1^ withanolides. (**c**) Kaplan–Meier survival plot of HV larvae fed on (i) 5 × 10^5^ CFU per ml Bt spores, (ii) 5 × 10^5^ CFU per ml Bt spores and 100 μg ml^−1^ withanolides and (iii) 5 × 10^5^ CFU per ml Bt spores and 150 μg ml^−1^ withanolides. Significant differences are indicated by ***P<0.001; **P<0.01, NS, not significant (Cox regression survival analysis). See Supplementary Table 6 for significance values.

The survival rates of *H. subflexa* larvae fed on diet containing 100 or 150 μg ml^−1^ withanolides in addition to 5 × 10^5^ CFU per ml Bt spores were significantly higher than the survival rates of larvae fed on only Bt spore diet (Fig. 5b; Supplementary Table 6a; cox proportional hazard model, 100 μg ml^−1^ withanolides: Wald test=12.375, df=1, *P*<0.001; 150 μg ml^−1^ withanolides: Wald test=7.760, df=1, *P*=0.005). In *H. virescens* the survival rates were slightly reduced after feeding on Bt and withanolides compared with only Bt diet; however, this trend was not statistically significant (Fig. 5c; Supplementary Table 6b; cox proportional hazard model, 100 μg ml^−1^ withanolides: Wald test=2.301, df=1, *P*=0.129; 150 μg ml^−1^ withanolides: Wald test=0.0106, df=1, *P*=0.745).

Due to the high variance between larvae that were able to grow and those that did not grow (Supplementary Fig. 4), we divided the larvae into two categories: larvae with a positive or a negative weight gain. We then analysed whether the proportion of larvae with positive or negative weight gains depended on the treatment or the species. Overall, the treatments affected the two species differently (glm, interaction deviance=−16.536, df=2, *P*<0.001). While more *H. subflexa* larvae grew with withanolide supplement, more *H. virescens* larvae grew without withanolides (Fig. 5a). The proportion of larvae with a positive weight gain was not influenced by the treatment (glm, Bt: 32.6%, Bt w100: 21.7%, Bt w150: 23.4%, (deviance=−1.436, df=2, *P*=0.488), although a higher proportion of *H. virescens* than *H. subflexa* larvae were able to grow (glm, 37.7% versus 14.3%, deviance=−10.178, df=1, *P*<0.001).

## Discussion

Withanolides, the major secondary plant compounds in the genus *Physalis*, have harmful effects on every biological system tested to date. Our study demonstrates the first beneficial effects of withanolides in any insect species. Withanolides have a positive impact on the specialist *H. subflexa* larvae that feed exclusively on *Physalis* plants by increasing larval growth and immune system activity, in contrast to its close relative *H. virescens*, which does not experience advantageous properties from a withanolide diet. Moreover, feeding on withanolide-supplemented diet improved the survival rate of *H. subflexa* larvae infected with Bt, suggesting that withanolides have played an important role in the evolution of *H. subflexa*'s adaptation to its host plant.

Withanolides have numerous well-documented harmful effects on diverse biological systems. They have feeding deterrent and immunosuppressive effects on several insect species^22^, including at least one heliothine, *Helicoverpa zea*^34^. They also trigger apoptosis, a programmed cell death pathway shared by all animals^35^,^36^. Withanolides are phytoecdysteroids, which are plant-produced compounds that have been shown to antagonize ecdysteroid activity in insects^37^. Ecdysteroid metabolism is known to control insect development, and disruption of this metabolism compromises insect development. Furthermore, ecdysteroids were shown to modulate cellular and humoral immunity in *Drosophila melanogaster* by increasing phagocytic activity, pathogen encapsulation and AMP gene expression^38^,^39^,^40^. Withanolides have been shown to have antagonistic ecdysteroid activity in *D. melanogaster* cell lines^24^,^41^. The feeding deterrent and growth-inhibiting effect of withanolides on most insects might therefore arise from the disruptive impact of these compounds on insect development and immunity. We found feeding deterrent and growth inhibitory effects of withanolides in the generalist moth species *H. virescens*. In contrast, withanolides positively affected the relative weight gain of the closely related specialist moth species *H. subflexa* when the caterpillars were fed diet containing biologically relevant concentrations of withanolides^42^. Thus, *H. subflexa* might have evolved mechanisms to prevent the effect of withanolides on their ecdysteroid receptors and thus avoid the negative effects of the phytochemical on insect development and immunity.

The gut microbiota in herbivorous insects can play an important role in nutrition and digestion. In general, food-derived phytochemicals are assumed to change the composition of the bacterial gut microbiota^43^,^44^. Since withanolides possess antibacterial properties^19^,^26^, they potentially affect the composition of larval gut microbiota. For example, in *Rhodnius prolixus,* a blood-sucking bug and an important vector of the Chagas disease, withanolide treatment resulted in higher numbers of bacterial microbiota but also in a lower antibacterial activity^45^. The fact that we found an increase in the relative weight gain in *H. subflexa* larvae feeding on diet containing withanolides suggests that withanolides have a nutritional or digestive microbial-driven effect, which reached its full potential at a concentration of 100 μg of withanolides in our experiments (Fig. 1a). Thus, withanolides may favour specific bacterial strains that promote nutrition and digestion in the specialist *H. subflexa*, or *H. subflexa* might be able to overcome limitations in the composition of its gut microbiota with the help of withanolides.

Previous studies have shown that withanolides have a potent immunosuppressive effect on other insects, inhibiting antibacterial activity, phagocytosis and hemocytic proliferation, and on human cells, suppressing T- and B-cell activation and proliferation^22^,^23^,^46^. These effects may result from the ecdysteroid-modulating properties of withanolides discussed earlier. Such an immune inhibitory effect of withanolides was not evident in the specialized *H. subflexa* larvae. Instead, their immune system appeared to be stimulated, with a significant increase of the phenoloxidase activity, while in *H. virescens* the PO activity was unaffected by withanolide supplementation (Fig. 1b). Phenoloxidase catalyses early steps in melanin formation and is a key factor of the immune response in invertebrates, as it is involved in cellular defence, haemolytic clotting and wound repair^47^. We also observed a non-significant increase in general antibacterial activity in the *H. subflexa* haemolymph after larvae ingested diet-containing withanolides (Supplementary Fig. 3). The stimulatory effect of withanolides on the *H. subflexa* immune system is further supported by our transcript data (Fig. 2), as we found that most of the identified immune genes in *H. subflexa* were upregulated after larvae had ingested withanolides.

In accordance with studies on other insect species^21^,^48^,^49^,^50^, we found the relative weight gain of *H. virescens* to be significantly affected by withanolides supplementation and the immune-related genes of *H. virescens* to be downregulated by withanolides (Fig. 2). Phenoloxidase activity and general immune activity in the haemolymph on the other hand were unaffected by withanolide ingestion rather than downregulated. These divergent results might be a consequence of the RNAseq data reflecting changes in the whole larval gut, while the haemolymph measurements only give a report of the haemolymph immune status. Importantly, *H. virescens* did not show the same immune-stimulating effects by withanolides as *H. subflexa,* suggesting that the immune stimulation is a direct effect of host plant adaptation by *H. subflexa*.

The evolution of plant defence is driven not only by herbivorous insects, but to a large extent also by microorganisms^51^. Many antibacterial agents in nature are secondary metabolites of plants^52^,^53^ and some have been shown to prevent insect infection by bacteria or viruses^54^,^55^. Withanolides are known to be active on a broad range of Gram-negative and Gram-positive bacteria relevant in human pathology^19^,^26^. However, the antibacterial activity of withanolides has not yet been investigated in an ecologically relevant context. We found that vegetative cells and spores of the microorganism *B. thuringiensis*, which are toxic to many insects^28^,^29^, were susceptible to extracts from *P. peruviana* containing withanolides. The antibacterial activity of *Physalis* plants may have evolved as a defence mechanism against bacterial plant pathogens, with the antibacterial activity of withanolides against the insect pathogen *B. thuringiensis* occurring as a side effect.

The antibacterial mode of action of withanolides is not yet clear. However, the susceptibility to withanolides and plant extracts of *P. peruvivana* may be restricted to the class of Gram-positive bacteria, as it only showed inhibitory activity against Gram-positive bacteria in our experiments (Supplementary Fig. 5; Supplementary Tables 1 and 2). A triterpenoid from the plant *Maytenus blepharodes*, structurally related to withanolides, was found to inhibit cell wall synthesis and macromolecule synthesis of *B. subtilis*, and withanolides might act in a similar manner^56^.

The Bt toxins that are produced by Bt spores are toxic to certain insects^28^. In *H. subflexa* we found that withanolides reduced the larval mortality when exposed to Bt and increased the larval growth of Bt infected *H. subflexa* larvae (Fig. 5). The close relative *H. virescens* did not experience such benefits from withanolide consumption. The mortality of *H. virescens* larvae was slightly reduced when withanolides were consumed in addition to Bt spores and the relative weight gain drastically reduced on withanolide supplemented diet compared with a Bt-only diet. Our results thus suggest that *H. subflexa,* which is able to tolerate withanolides in high amounts, gain protection from entomopathogenic bacteria such as Bt by feeding on *Physalis*.

As the toxin produced by the Bt spores, rather than the Bt bacteria or spores themselves, is the main acute cause of insect death^28^, the antibacterial activity of withanolides against Bt may only partly explain the observed increased survival rates among *H. subflexa*. Withanolides might inhibit Bt bacteria from multiplying further, but the toxin in ingested spores would still be effective. However, the larvae might profit from the immune-stimulating effect of withanolides when challenged by Bt, as it is the case in *Ephestia kuehniella,* the flour moth, which has an increased survival rate on low Bt concentrations, when immune-stimulated^57^. For example, withanolides might enhance the regenerative capacity of the gut cells after Bt toxins are released in the larval gut lumen where the toxins induce cell lysis. In some insects, septicaemia caused by other midgut bacteria invading the haemolymph after the ingestion of Bt toxins has been suggested to be the major cause of mortality^58^. The antibacterial effect of withanolides might keep bacteria from invading the haemocoel and thereby suppress septicaemia. Thus, while the precise mechanisms by which withanolides increase the survival of *H. subflexa* feeding on Bt spores remain unknown, it has to be a specific interaction between *H. subflexa* and withanolides as the close relative *H. virescens* does not experience such advantages from feeding on withanolide supplemented Bt diet. Our results confirm previous work showing that the host plant cannot be ignored in assessing Bt toxicity in insects^59^.

Withanolides are found in many genera of Solanaceae, but also in other plant families^19^. Their toxic effects on animal systems suggest an adaptive benefit in reducing herbivory, but the coevolutionary aspects of plant–insect interactions have hardly been investigated for this class of compounds. Other insect species such as the false potato beetle have adapted to *Physalis* and presumably overcome the toxic effects of withanolides^60^. It would be interesting to see whether there are reciprocal adaptations and counter-adaptations by withanolide-producing plants; such has been documented for crucifers and certain crucifer-feeding specialists^61^,^62^.

Is *H. subflexa* now dependent on *Physalis* for optimal immune system function? In a previous study we found that *H. subflexa*, which had been reared on artificial diet without withanolides were less tolerant to Bt bacteria and had a less efficient cell-mediated immune response compared to *H. virescens*.^30^. In the current study, we found the opposite effect when both species are exposed to withanolides. In the evolutionary divergence of these closely related species about two and a half million years ago^10^, immune competence has evidently also diverged. Although the host range of the common ancestor is unknown, it is likely that *H. virescens* or its ancestor evolved a more efficient immune response as its host range increased, exposing it to a greater variety of pathogens, in accordance with the predictions of ecological immunology^30^. However, it is also likely that the immune system in the *H. subflexa* lineage evolved as it developed its unique specialization on *Physalis*: it not only overcame the inhibitory effect of withanolides on its immune system, but also evolved the ability to be stimulated by withanolides, possibly by circumventing ecdysteroid antagonistic activities. In adapting to withanolides, *H. subflexa* has likely become partially dependent on them for its immune system to function optimally. This dependence may be an unexpected corollary of the dictum that evolutionary specialization is a dead end.

In conclusion, withanolides derived from the host plant directly and indirectly protect *H. subflexa* from bacterial pathogens. Withanolides actively inhibit Bt spore germination and vegetative cell growth and thereby reduce pathogen loads for *H. subflexa*. Withanolides also stimulate the immune response of *H. subflexa*, opposite to the effect these compounds have on all other insects studied to date, including its close relative *H. virescens*. This indirect effect of withanolides further enables *H. subflexa* larvae to defend themselves against bacterial pathogens. Hence, a consequence of *H. subflexa* specialization on *Physalis* plants is the adaptation of its immune system to the impact of withanolides, enabling *H. subflexa* to exploit their positive antibacterial properties. Beyond simply countering the plant-produced anti-herbivore compounds or using them for its own defenses (for example, by sequestration), the insect has succeeded in converting the inhibitory effects of withanolides into activation effects for its own advantage, demonstrating a unique benefit to host–plant specialization.

## Methods

### Insects

*Heliothis subflexa* collected near Gainesville, Florida was provided by the USDA Insect Attractants, Behavior and Basic Biology Research Laboratory and reared at North Carolina State University (NCSU) from 1989 on a corn–soy blend-based artificial diet (80 g agar; 700 g corn soy blend; 36.5 g whole milk powder; 40 g torula yeast; 4 g sorbic acid; 8 g methyl paraben; 15 g vitamin mix; 14 g ascorbic acid per 3.7 l of water) until transfer to Jena in 2006. *Heliothis virescens* collected near Clayton, North Carolina was provided by NCSU and reared on a pinto-bean based artificial diet (35 g agar; 125 g pinto bean; 100 g wheat germ; 50 g soy protein; 50 g casein; 62,5 g torula yeast; 10 g vanderzant vitamin mixture; 6 g ascorbic acid; 5 g methyl paraben; 3 g sorbic acid; 0.25 g tetracycline per 1.1 l of water). Larvae of both species were held in a climate chamber (Snijder) at 26 °C, 55±10% relative humidity and 16 h/8 h light–dark. Third-instar larvae were used in all experiments. All larval experiments were performed in 24-well polystyrene plates (VWR International, Darmstadt, Germany) kept together in a clip box; each well contained one larva on 1 ml artificial diet.

### Preparation of crude leaf homogenates and purified withanolides

Plant material from *Physalis peruviana*, *Brassica napus* (rapeseed) and *Solanum tuberosum* (potato) were collected from greenhouse plants. Leaves and calyx were crushed in a 1.5 ml tube with the help of a plastic pestle and the resulting liquid (referred to as crude leaf homogenates) was directly used for inhibition zone assays.

To obtain purified withanolides, aerial parts (leaves and fruits) of *P. peruviana* were collected from greenhouse plants, frozen at −20 °C and dried in a lyophilisator at −80 °C for 2 days. The dry plant material (5 g) was extracted three times with MeOH (2 l) at room temperature to obtain a crude extract. This crude extract contains withanolides and flavonoids and was used in inhibition zone assays as a negative control (referred to as less purified methanolic leaf extract). To separate withanolides, the volume of the crude extract was reduced to ∼400 ml, 100 ml H_2_O was added and subjected to PS/DVB SPE cartridges (Chromabond HR-X, Macherey & Nagel) according to standard protocol. The cartridges were eluted with acetone and collected, dried, and diluted before use. Analyses were carried out using a Finnigan LTQ mass spectrometer (Thermo Electron Corp., Im Steingrund 4–6, 63303 Dreieich, Germany) in the APCI-positive mode (vaporizer temperature: 450 °C, capillary temperature 275 °C) connected to an Agilent HP1100 HPLC system equipped with an RP18 column, LiChroCART (250 × 4 mm, 5 μm; Merck KGaA, 64271, Darmstadt, Germany). Samples were analysed by using gradient elution at 1 ml min^−1^ (solvent A: H_2_O+0.5% CH3COOH; solvent B: MeCN+0.1% CH_3_COOH) according to the following protocol: starting with 20% B, holding for 5 min, going to 100% B in 30 min, with subsequent washing.

Substances were tentatively identified as steroid lactones based on characteristic fragmentation patterns, such as multiple loss of water and loss of the lactone moiety^63^.

### Feeding assays

To measure the impact of withanolides on *Heliothis subflexa* and *H. virescens* larvae, purified withanolide extracts were added to 1 ml artificial diet within 24-well polystyrene plates (Supplementary Fig. 8). All applied withanolide concentrations correspond to naturally occurring withanolide concentrations in the berry of *Physalis peruviana*^17^. To incorporate these compounds into the artificial diet, purified withanolideswere dissolved in 40% methanol and applied to the diet. Afterwards the 24-well plates were placed under a fume cupboard to allow the solvent to evaporate. Early third-instar larvae were placed separately in a well containing 1 ml diet incorporated with 10, 25, 50, 100, 150 or 200 μg of withanolides, reflecting low up to natural concentrations in *Physalis peruviana*^17^. For each treatment, 24 larvae were tested for 7 days, and on every second day larvae were provided with freshly prepared diet containing withanolides to ensure the activity of the compounds. In the control treatment, larvae were reared on artificial diet containing the evaporated pure solvent (40% methanol). Larval weight was recorded daily for seven days. The relative weight gain was estimated as followed:

Relative weight gain [mg]=(highest weight [mg]−start weight [mg])/start weight [mg])

To investigate the effect of *B. thuringiensis* HD73 (Bt) spores on *H. subflexa* larvae, Bt spores were added to 1 ml artificial diet within 24-well polystyrene plates. Early third-instar larvae were exposed to diets containing 10^3^, 10^4^, 5 × 10^4^, 7.5 × 10^4^, 10^5^, 5 × 10^5^ or 10^6^ CFU per ml Bt spores (see Supplementary Methods for spore production). For each treatment, the spore solution was applied to 1 ml diet. For seven days, larval growth and survival rates of 24 larvae per treatment were recorded. On every second day larvae were provided with freshly prepared diet containing Bt spores to ensure the activity of the compounds. In the control treatment, larvae were reared on pure artificial diet.

To determine the impact of withanolides on Bt spore infected *H. subflexa* and *H. virescens* larvae, an experiment combining feeding Bt spores and withanolides was performed. First of all, 100 μg ml^−1^ or 150 μg ml^−1^ purified withanolides diluted in 40% methanol were applied to 1 ml artificial diet. The 24-well plates were placed under a fume cupboard to allow the solvent to evaporate. After the solvent was evaporated, the lethal Bt spore concentration of 5 × 10^5^ CFU per ml was added in each well containing artificial diet incorporated with withanolides. As soon as the Bt spore solution was soaked in the diet, early third-instar larvae were separated in each well. In the control treatment, larvae were reared on artificial diet containing only 5 × 10^5^ CFU per ml Bt spores. For each treatment, the larval growth and survival rates of 24 larvae per treatment were recorded. On every second day larvae were provided with freshly prepared diet containing Bt spores and withanolides to ensure the activity of the compounds For all experiments, the larvae in the 24-well plates were kept together in a clip box in the climate chamber, as described above.

The Bt spore dose of 5 × 10^5^ CFU per ml used here was experimentally determined as the minimal dose that would still kill most of the population. Whether this dose is similar to a dose *H. subflexa* and *H. virescens* might encounter in a natural environment is difficult to evaluate. The few attempts to measure the density of Bt spores in nature^64^,^65^,^66^ have documented a very patchy distribution.

### Antibacterial activity of crude plant homogenates and withanolides

The antibacterial activity of crude plant homogenates from *Physalis peruviana* calyx and leaves (Solanaceae), potato leaves (Solanaceae) and rapeseed leaves (Brassicaceae) were tested against selected bacteria (Supplementary Fig. 5). *Bacillus thuringiensis* subsp. *kurstaki* strain HD 73 (Bt), *Bacillus subtilis*, *Pseudomonas putida*, *Escherichia coli* (strain DH5alpha), *Serratia entomophila* and *Lactobacillus casei*, which were obtained from the Department of Bioorganic Chemistry (MPICE, Jena, Germany) were used in all inhibition zone assays. To obtain vegetative cells, all bacterial strains were cultured at 30 °C and 250 r.p.m. in lysogeny broth (LB) or on LB agar^67^, except for *S. entomophila* which was grown in CASO medium^68^ and *E. coli* which was grown in LB medium at 37 °C. Bacterial cells were obtained from overnight cultures and cell counts were estimated by optical density at 600 nm (BioPhotometer, Eppendorf, Hamburg, Germany). Beforehand, known numbers of CFU were plotted against their corresponding optical density at 600 nm to obtain a standard curve for bacterial cell concentration of each strain. Besides vegetative bacterial cells, spores of Bt (see Supplementary Methods for spore production) were also used for inhibition zone assays. For all assays, an aliquot of 500 μl vegetative bacterial cultures or 25 μl spores of Bt (each of which contains approximately 4 × 10^9^ CFU per ml) was added to 500 ml LB medium and plates were poured (10 ml per plate ). Holes within the Petri dish were made by puncturing the agar with a plastic pipette (Eppendorf Research 5000) and removing the agar plug by suction. Crude leaf homogenates (2 μl) were placed in each well and the plates were incubated for 24 h at 30 °C and the antibacterial activity was determined as the radius of the clear zone around a sample well (Supplementary Fig. 5). All assays were done in triplicates to verify the results.

We further investigated the antibacterial activity of purified withanolides of *P. peruviana* against spores of Bt. Therefore, 2 μl of 10 μg μl^−1^, 20 μg μl^−1^ and 30 μg μl^−1^ withanolides were placed into the holes of the agar plates that contain Bt spores. As controls, less purified methanolic leaf extracts (containing withanolides and flavonoids), crude *P. peruviana* leaf homogenates, methanol and gentamycin were tested for their antibacterial activity. After 24 h of incubation at 30 °C, the antibacterial activity was determined as the radius of the clear zone around a sample well (Fig. 3a,b).

To reproduce the conditions on *Physalis* plants, we inoculated 10 ml LB agar with 100 μg ml^−1^ withanolides. As a negative control, agar plates were inoculated with the solvent 40% methanol. After the agar plates were dried, an aliquot (10 μl; 4 × 10^3^ CFU ml^−1^) from vegetative cells and spores of Bt was spread on the agar plates. After 48 h of incubation at 30 °C, CFU were counted (Fig. 3c,d).

To obtain a gentamycin dilution series and subsequently a standard curve, inhibition zones on LB agar inoculated with Bt spores, were measured and repeated in triplicate. Two μl of water containing 0.06, 0.15, 0.25, 0.5, 1 and 2 μg μl^−1^ gentamycin were placed in the holes punctured with a pipette tip on agar plates. The obtained average inhibition zone was plotted in a graph to which a logarithmic regression line was fitted (Supplementary Fig. 9). To quantify withanolide activity in relation to gentamycin activity, the average inhibition zone of withanolides was used as *y* in the following equation, in which *x* stands for the corresponding gentamycin concentration *x*=*e*^((*y*−8.9)/2.8)^ *y*. The results are given in Supplementary Table 7.

As high concentrations of isothiocyanates (ITCs) have been shown to have antimicrobial activity^69^,^70^, we estimated the amounts of ITCs likely to be present in leaf homogenates of *B. napus*, in which active myrosinase was likely present, hydrolysing the glucosinolates to ITCs. Cole 1976 (ref. 71) estimated the total amounts of ITCs after autolysis in *B. napus* to be about 4 μg g^−1^. This converts to 0.01 μmol ITC per g plant fresh weight, if we use the molecular mass of sinigrin (297.47 g mol^−1^). Tierens *et al*.^70^ found ITCs to be active against *E. coli* at a concentration of 0.282 μmol g^−1^, nearly 30 times higher than would be produced in the *B. napus* crude leaf homogenate that we used.

### Phenoloxidase activity and antibacterial activity in the larval haemolymph

To compare phenoloxidase (PO) activity in the haemolymph of larvae fed on artificial diet and larvae fed on artificial diet containing 100 μg ml^−1^ withanolides as described above, a PO activity assay was performed. Forty-two larvae per treatment were assessed for their PO activity. Haemolymph was extracted after 7 days of treatment by puncturing the larvae with a sterile hypodermic needle. Ten microlitre haemolymph of each larva was collected separately, 500 μl of ice-cold sodium cacodylate buffer (0.01 M Na–cacodylate, 0.005 M CaCl_2_) was added and the mixture directly frozen in liquid N_2_ and stored at −80 °C. To measure PO activity, frozen samples were thawed at room temperature then centrifuged at 4 °C and 2800, g for 15 min. Subsequently, 100 μl of the resulting supernatant was transferred to a 96-well polystyrene plate (VWR International, Darmstadt, Germany) containing 200 μl of 3 mM L-Dopa (Sigma) per single well, and absorbance was measured for 45 min at 490 nm and 30 °C, taking absorbance measurements once every minute. (Multiskan Spectrum multiplate reader; Thermo-Electron). The fastest change in absorbance, from 15–26 min (*v*_max_) of the reaction, was used which is within the linear range of 5–45 min after adding the substrate. Data were obtained with SkanIt Software for Multiskan Spectrum version 2.1 (Thermo-Electron).

To measure antibacterial activity of larval haemolymph in response to exposure with 100 μg ml^−1^ withanolides, an inhibition zone assay was performed. Haemolymph samples were extracted from larvae after 7 days of treatment as described above. An aliquot (500 μl) of an overnight culture of *E. coli* (strain DH5alpha) was added to 500 ml LB medium. Petri dishes were filled with 10 ml LB medium containing *N*-phenylthiourea to inhibit the melanization of the haemolymph samples. Holes within the Petri dish were made by puncturing the agar with a plastic pipette (Eppendorf Research 5000) and removing the agar plug by suction. Two microlitre of undiluted haemolymph samples from 42 individual larvae were placed into the holes of the agar plate. As a positive control, chicken egg white lysozyme at concentrations between 2 and 0.03 μg μl^−1^ was used, and a calibration curve was created based on these standards. After 24 h of incubation at 37 °C, the antibacterial activity was determined as the radius of the clear zone around a sample well.

### Transcriptome sequencing and annotation of the assembly

To find differentially expressed immune-related genes between *H. subflexa* and *H. virescens* in response to withanolides, larvae were fed on artificial diet and on artificial diet containing 100 μg ml^−1^ withanolides as described above. After three days, the gut was isolated from the rest of the body and RNA was extracted. Each RNA pool contained three larvae guts. For each species two pools per treatment were sequenced, resulting in a total of eight sequenced RNA pools (see Supplementary Methods for detailed description of RNA isolation and transcriptome sequencing, assembly and annotation and Supplementary Methods for detailed description of remapping and digital expression analysis).

### Data analysis

To test for the effect of withanolides and Bt supplementation on the relative weight gain of the larvae as well as on the PO activity of the larval haemolymph, a generalized least squares method^72^ was used to account for the heterogeneity of the data. The varIdent variance structure was applied to allow different spread for each treatment group. *P* values were obtained by removing non-significant variables and comparison of models with a likelihood ratio test^73^. To identify treatments that are different to each other factor level reduction was applied^74^. The analyses were done in R 3.2.0 (ref. 75).

To test the influence of the withanolide treatment on the number of samples showing an antibacterial activity we used generalized linear model for presence—absence data (glm, with the binomial error family). *P* values were obtained by deleting the explanatory variables and subsequent comparison of the more complex model with the simpler model^73^. The influence of the withanolide treatment on the strength of the antibacterial activity of the haemolymph of the two species was tested with a two-way ANOVA.

Larval survival experiments were analysed using the Cox proportional hazard model to test the effect of the treatment on the survival of larvae post exposure. To illustrate the effect of treatment on the survival of both species, we used the Kaplan–Meier survivorship function. These statistical tests were run with the computer program SPSS 17.0.

The results of the inhibition assays were analysed by ANOVA, with the different bacteria strains and the different crude leaf homogenates, purified withanolides and gentamycin treatments as fixed effects. After ANOVAs, contrast tests between controls (potato and rapeseed seed leaves) and *Physalis* and withanolide treatments were performed as orthogonal planned comparisons. Statistical analysis of colony numbers on withanolides and methanol plates was done using an unpaired *t*-test. All the statistical analyses were performed using JMP 4 (SAS Institute).

To test influence of the Bt treatment and Bt treatment with withanolide supplementation on *H. virescens* and *H. subflexa* we divided the larvae in two categories: larve with a positive relative growth rate and larvae with a negative relative growth rate. To test whether the amount of larvae with a positive growth rate was influenced by the applied treatment a generalized linear model for presence—absence data (glm, with the binomial error family) was used. *P* values were obtained by deleting the explanatory variables and subsequent comparison of the more complex model with the simpler model^73^. The analyses were done in R 3.2.0 (ref. 75).

### Data availability

We have deposited the short-read (Illumina HiSeq2500) data with the following study accession number: PRJEB8293 (EBI short-read archive). The complete study is also accessible directly at the following URL: http://www.ebi.ac.uk/ena/data/view/PRJEB8293. The complete set of *H. virescens* and *H. subflexa* AMPs with contig consensus sequences, Blast2GO hits against the NR database, hit accessions, putative annotations and relative expression levels across the different RNAseq samples are in Supplementary Data 1. Other data are available from the authors on request.

## Additional information

**How to cite this article**: Barthel, A. *et al*. Immune modulation enables a specialist insect to benefit from antibacterial withanolides in its host plant. *Nat. Commun.* 7:12530 doi: 10.1038/ncomms12530 (2016).

## Supplementary Material

Supplementary Information Supplementary Figures 1-9, Supplementary Tables 1-7, Supplementary Methods and Supplementary References

Supplementary Data 1 List of differently expressed genes from RNAseq analysis

## References

[b1] Ehrlich P. R. & Raven P. H. Butterflies and plants: a study in coevolution. Evolution 18, 586–608 (1964).

[b2] Fox L. R. Defense and dynamics in plant-herbivore systems. Am. Zool. 21, 853–864 (1981).

[b3] Jaenike J. Host specialization in phytophagous insects. Annu. Rev. Ecol. Syst. 21, 243–273 (1990).

[b4] Duffey S. S. Sequestration of plant natural products by insects. Annu. Rev. Entomol. 25, 447–477 (1980).

[b5] Ali J. G. & Agrawal A. A. Specialist versus generalist insect herbivores and plant defense. Trends Plant Sci. 17, 293–302 (2012). 22425020 10.1016/j.tplants.2012.02.006

[b6] Petschenka G., Pick C., Wagschal V. & Dobler S. Functional evidence for physiological mechanisms to circumvent neurotoxicity of cardenolides in an adapted and a non-adapted hawk-moth species. Proc. Biol. Sci. 280, 20123089 (2013). 23516239 10.1098/rspb.2012.3089 PMC3619502

[b7] Kirsch R. . Host plant shifts affect a major defense enzyme in *Chrysomela lapponica*. Proc. Natl Acad. Sci. USA 108, 4897–4901 (2011). 21383196 10.1073/pnas.1013846108 PMC3064323

[b8] Richards L. A. . Synergistic effects of iridoid glycosides on the survival, development and immune response of a specialist caterpillar, Junonia coenia (Nymphalidae). J. Chem. Ecol. 38, 1276–1284 (2012). 23053916 10.1007/s10886-012-0190-y

[b9] Brazell J. R. . Bollworm and tobacco budworm as cotton pests in Louisiana and Arkansas. Louisiana Agric. Exp. Stat. Tech. Bull. 482, art. 47 (1953).

[b10] Cho S. . Molecular phylogenetics of heliothine moths (Lepidoptera: Noctuidae: Heliothinae), with comments on the evolution of host range and pest status. Syst. Entomol. 33, 581–594 (2008).

[b11] Petzold J., Brownie C. & Gould F. Effect of *Heliothis subflexa* herbivory on fruit abscission by *Physalis* species: the roles of mechanical damage and chemical factors. Ecol. Entomol. 34, 603–613 (2009).

[b12] Hardwick D. F. A Monograph to the North American Heliothentinae (Lepidoptera: Noctuidae) Agriculture Canada (1996).

[b13] Mitter C., Poole R. W. & Matthews M. Biosystematics of the Heliothinae (Lepidoptera, Noctuidae). Annu. Rev. Entomol. 38, 207–225 (1993).

[b14] Poole R. W., Mitter C. & Huettel M. A Revision and Cladistic Analysis of the Heliothis virescens Species-Group (Lepidoptera: Noctuidae) with a Preliminary Morphometric Analysis of Heliothis virescens Mississippi Agriculture & Forestry Experiment Station Technical Bulletin. 185, 1–51 ((1993).

[b15] Oppenheim S. J. & Gould F. Behavioral adaptations increase the value of enemy-free space for *Heliothis subflexa*, a specialist herbivore. Evolution 56, 679–689 (2002). 12038526 10.1111/j.0014-3820.2002.tb01379.x

[b16] Sisterson M. S. & Gould F. L. The inflated calyx of *Physalis angulata*: A refuge from parasitism for *Heliothis subflexa*. Ecology 80, 1071–1075 (1999).

[b17] Baumann T. W. & Meier C. M. Chemical Defense by withanolids during fruit development in *Physalis peruviana*. Phytochemistry 33, 317–321 (1993).

[b18] Ramadan M. F. Bioactive phytochemicals, nutritional value, and functional properties of cape gooseberry (*Physalis peruviana*): An overview. Food Res. Int. 44, 1830–1836 (2011).

[b19] Misico R. I. . in Progress in the Chemistry of Organic Natural Products Vol. 94, eds Kinghorn A. D., Falk H., Kobayashi J. 127–229Springer (2011).21833839

[b20] Ascher K. R. S. . Insect antifeedant properties of withanolides and related steroids from Solanaceae. Experientia 36, 998–999 (1980).

[b21] Bado S., Mareggiani G., Amiano N., Burton G. & Veleiro A. S. Lethal and sublethal effects of withanolides from Salpichroa origanifolia and analogues on Ceratitis capitata. J. Agric. Food Chem. 52, 2875–2878 (2004). 15137828 10.1021/jf035508a

[b22] Castro D. P. . Immune depression in *Rhodnius prolixus* by seco-steroids, physalins. J. Insect Physiol. 54, 555–562 (2008). 18234209 10.1016/j.jinsphys.2007.12.004

[b23] Yu Y. . Investigation of the immunosuppressive activity of Physalin H on T lymphocytes. Int. Immunopharmacol. 10, 290–297 (2010). 19951747 10.1016/j.intimp.2009.11.013

[b24] Dinan L., Whiting P., Alfonso D. & Kapetanidis I. Certain withanolides from *Lochroma gesnerioides* antagonize ecdysteroid action in a *Drosophila melanogaster* cell line. Entomol. Exp. Appl. 80, 415–420 (1996).

[b25] Lan Y. H. . New cytotoxic withanolides from *Physalis peruviana*. Food Chem. 116, 462–469 (2009).

[b26] Silva M. T. G., Simas S. M., Batista T., Cardarelli P. & Tomassini T. C. B. Studies on antimicrobial activity, *in vitro*, of *Physalis angulata* L. (Solanaceae) fraction and physalin B bringing out the importance of assay determination. Mem. Inst. Oswaldo Cruz 100, 779–782 (2005). 16410969 10.1590/s0074-02762005000700018

[b27] Misico R. I. . in Progress in the Chemistry of Organic Natural Products Vol. 94, eds Kinghorn A. D., Falk H., Kobayashi J. 127–229 ((2011).21833839

[b28] Schnepf E. . *Bacillus thuringiensis* and its pesticidal crystal proteins. Microbiol. Mol. Biol. Rev. 62, 775–806 (1998). 9729609 10.1128/mmbr.62.3.775-806.1998 PMC98934

[b29] Estruch J. J. . Vip3A, a novel *Bacillus thuringiensis* vegetative insecticidal protein with a wide spectrum of activities against lepidopteran insects. Proc. Natl Acad. Sci. USA 93, 5389–5394 (1996). 8643585 10.1073/pnas.93.11.5389 PMC39256

[b30] Barthel A. . Immune defence strategies of generalist and specialist insect herbivores. Proc. R. Soc. B Biol. Sci. 281, 20140897 (2014). 10.1098/rspb.2014.0897 PMC4083799 24943370

[b31] Rolff J. & Siva-Jothy M. T. Invertebrate ecological immunology. Science 301, 472–475 (2003). 12881560 10.1126/science.1080623

[b32] Schulenburg H., Kurtz J., Moret Y. & Siva-Jothy M. T. Ecological immunology. Phil. Trans. R. Soc. B Biol. Sci. 364, 3–14 (2009). 10.1098/rstb.2008.0249 PMC2666701 18926970

[b33] Berenbaum M. R. in Novel Aspects of Insect-Plant Interactions eds Barbosa P., LeTourneau D. K. 97–123Wiley (1988).

[b34] Waiss A. C., Elliger C. A., Haddon W. F. & Benson M. Insect inhibitory steroidal saccharide esters from *Physalis peruviana*. J. Nat. Prod. 56, 1365–1372 (1993).

[b35] Grogan P. T., Sarkaria J. N., Timmermann B. N. & Cohen M. S. Oxidative cytotoxic agent withaferin A resensitizes temozolomide-resistant glioblastomas via MGMT depletion and induces apoptosis through Akt/mTOR pathway inhibitory modulation. Invest. New Drugs 32, 604–617 (2014). 24718901 10.1007/s10637-014-0084-7 PMC4380174

[b36] Vandenberghe I. . Physalin B, a novel inhibitor of the ubiquitin-proteasome pathway, triggers NOXA-associated apoptosis. Biochem. Pharmacol. 76, 453–462 (2008). 18577376 10.1016/j.bcp.2008.05.031

[b37] Dinan L. Phytoecdysteroids: biological aspects. Phytochemistry 57, 325–339 (2001). 11393511 10.1016/s0031-9422(01)00078-4

[b38] Lanot R., Zachary D., Holder F. & Meister M. Postembryonic hematopoiesis in *Drosophila*. Dev. Biol. 230, 243–257 (2001). 11161576 10.1006/dbio.2000.0123

[b39] Sorrentino R. P., Carton Y. & Govind S. Cellular immune response to parasite infection in the *Drosophila* lymph gland is developmentally regulated. Dev. Biol. 243, 65–80 (2002). 11846478 10.1006/dbio.2001.0542

[b40] Meister M. & Richards G. Ecdysone and insect immunity: the maturation of the inducibility of the diptericin gene in *Drosophila* larvae. Insect Biochem. Mol. Biol. 26, 155–160 (1996). 8882658 10.1016/0965-1748(95)00076-3

[b41] Gilbert L. I., Bollenbacher W. E. & Granger N. A. Insect endocrinology-regulation of endocrine glands, hormone titer, and hormone metabolism. Annu. Rev. Physiol. 42, 493–510 (1980). 6996594 10.1146/annurev.ph.42.030180.002425

[b42] Metcalf R. L., Metcalf R. A. & Rhodes A. M. Cucurbitacins as kairomones for diabroticite beetles. Proc. Natl Acad. Sci. USA 77, 3769–3772 (1980). 16592849 10.1073/pnas.77.7.3769 PMC349707

[b43] Felton G. W., Workman J. & Duffey S. S. Avoidance of antinutritive plant defense-role of midgut pH in Colorado potato beetle. J. Chem. Ecol. 18, 571–583 (1992). 24253867 10.1007/BF00987820

[b44] Priya N. G., Ojha A., Kajla M. K., Raj A. & Rajagopal R. Host plant induced variation in gut bacteria of *Helicoverpa armigera*. PLoS ONE 7, e30768 (2012). 22292034 10.1371/journal.pone.0030768 PMC3266921

[b45] Castro D. P. . Physalin B inhibits *Trypanosoma cruzi* infection in the gut of *Rhodnius prolixus* by affecting the immune system and microbiota. J. Insect Physiol. 58, 1620–1625 (2012). 23085484 10.1016/j.jinsphys.2012.10.001

[b46] Huang C.-F. . Immunosuppressive Withanolides from *Withania coagulans*. Chem. Biodivers. 6, 1415–1426 (2009). 19774603 10.1002/cbdv.200800211

[b47] Cerenius L. & Soderhall K. The prophenoloxidase-activating system in invertebrates. Immunol. Rev. 198, 116–126 (2004). 15199959 10.1111/j.0105-2896.2004.00116.x

[b48] Ascher K. R. S., Schmutterer H., Glotter E. & Kirson I. Withanolides and related ergostane-type steroids as antifeedants for larvae of *Epilachna varivestis* (Coleoptera, Chrysomelidae). Phytoparasitica 9, 197–205 (1981).

[b49] Ascher K. R. S. . The Antifeedant Effect of some new withanolides on 3 insect species, Spodoptera littoralis, Epilachna varivestis and Tribolium castaneum. Phytoparasitica 15, 15–29 (1987).

[b50] Mareggiani G. . Response of Tribolium castaneum (Coleoptera, Tenebrionidae) to Salpichroa origanifolia withanolides. J. Agric. Food Chem. 50, 104–107 (2002). 11754551 10.1021/jf010766y

[b51] Thompson J. N. The coevolutionary process Univ. Chicago Press (1994).

[b52] Krischik V. A., Barbosa P. & Reichelderfer C. F. 3 trophic level interactions-allelochemicals, *Manduca sexta* (L), and *Bacillus thuringiensis* var *kurstaki* Berliner. Environ. Entomol. 17, 476–482 (1988).

[b53] Chun S. S., Vattem D. A., Lin Y. T. & Shetty K. Phenolic antioxidants from clonal oregano (*Origanum vulgare*) with antimicrobial activity against *Helicobacter pylori*. Process Biochem. 40, 809–816 (2005).

[b54] Reichelderfer C. F. in Microbial Mediation of Plant-Herbivore Interactions eds Barbosa P., Krischik V. A., Jones C. G. 507–524Wiley (1991).

[b55] Felton G. W., Duffey S. S., Vail P. V., Kaya H. K. & Manning J. Interaction of nuclear polyhedrosis-virus with catechols-potential incompatibility for host-plant resistance against noctuid larvae. J. Chem. Ecol. 13, 947–957 (1987). 24302060 10.1007/BF01020174

[b56] de Leon L., Beltran B. & Moujir L. Antimicrobial activity of 6-oxophenolic triterpenoids. Mode of action against *Bacillus subtilis*. Plant Med. 71, 313–319 (2005). 10.1055/s-2005-864096 15856406

[b57] Rahman M. M., Roberts H. L. S., Sarjan M., Asgari S. & Schmidt O. Induction and transmission of *Bacillus thuringiensis* tolerance in the flour moth *Ephestia kuehniella*. Proc. Natl Acad. Sci. USA 101, 2696–2699 (2004). 14978282 10.1073/pnas.0306669101 PMC365683

[b58] Broderick N. A. . Contributions of gut bacteria to *Bacillus thuringiensis*-induced mortality vary across a range of Lepidoptera. BMC Biol. 7, art. 11 (2009). 10.1186/1741-7007-7-11 PMC2653032 19261175

[b59] Raymond B., Wright D. J. & Bonsall M. B. Effects of host plant and genetic background on the fitness costs of resistance to *Bacillus thuringiensis*. Heredity 106, 281–288 (2011). 20517345 10.1038/hdy.2010.65 PMC3044451

[b60] Gordh G. H., D A Dictionary of Entomology CSIRO Publishing (2011).

[b61] Edger P. P. . The butterfly plant arms-race escalated by gene and genome duplications. Proc. Natl Acad. Sci. USA 112, 8362–8366 (2015). 26100883 10.1073/pnas.1503926112 PMC4500235

[b62] Wheat C. W. . The genetic basis of a plant-insect coevolutionary key innovation. Proc. Natl Acad. Sci. USA 104, 20427–20431 (2007). 18077380 10.1073/pnas.0706229104 PMC2154447

[b63] Musharraf S. G. . Analysis and development of structure-fragmentation relationships in withanolides using an electrospray ionization quadropole time-of-flight tandem mass spectrometry hybrid instrument. Rapid Commun. Mass Spectrom. 25, 104–114 (2011). 21157859 10.1002/rcm.4835

[b64] Hastowo S., Lay B. W. & Ohba M. Naturally-occurring *Bacillus thuringiensis* in Indonesia. J. Appl. Bacteriol. 73, 108–113 (1992).

[b65] Ohba M. *Bacillus thuringiensis* populations naturally occurring on mulberry leaves: A possible source of the populations associated with silkworm-rearing insectaries. J. Appl. Bacteriol. 80, 56–64 (1996).

[b66] Rodríguez-Sánchez C., Sittenfeld A., Janzen D. H. & Espinoza A. M. *Bacillus thuringiensis* in caterpillars and associated materials collected from protected tropical forests in northwestern Costa Rica. Rev. Biol. Trop. 54, 265–271 (2006). 18494297 10.15517/rbt.v54i2.13867

[b67] Bertani G. Studies on lysogenesis. 1. The mode of phage liberation by lysogenic *Escherichia coli*. J. Bacteriol 62, 293–300 (1951). 14888646 10.1128/jb.62.3.293-300.1951 PMC386127

[b68] Trustees B. O. in The United States Pharmacopeial Convention United States Pharmacopeial Convention (1995).

[b69] Shofran B. G., Purrington S. T., Breidt F. & Fleming H. P. Antimicrobial properties of sinigrin and its hydrolysis products. J. Food Sci. 63, 621–624 (1998).

[b70] Tierens K. . Study of the role of antimicrobial glucosinolate-derived isothiocyanates in resistance of arabidopsis to microbial pathogens. Plant Physiol. 125, 1688–1699 (2001). 11299350 10.1104/pp.125.4.1688 PMC88826

[b71] Cole R. A. Isothiocyanates, nitriles and thiocyanates as products of autolysis of glucosinolates in cruciferae. Phytochemistry 15, 759–762 (1976).

[b72] Pinheiro J., Bates D., DebRoy S., Sarkar D. & R Core Team nlme: Linear and Nonlinear Mixed Effects Models. R package version 3.1-128 http://CRAN.R-project.org/package=nlme (2016).

[b73] Zuur A. F., Ieno E. N., Walker N. J., Saveliev A. A. & Smith G. M. Mixed Effects Models and Extensions in Ecology with R Springer (2009).

[b74] Crawley M. J. The R Book 1–942Wiley (2013).

[b75] R Core Team. R: A language and environment for statistical computing. (R Foundation for Statistical Computing, Vienna, Austria, 2014). http://www.R-project.org/.

