# Supplementary material for: Immune modulation enables a specialist insect to benefit from antibacterial withanolides in its host plant
Source: Nat Commun. 2016 Aug 26;7:12530. doi: 10.1038/ncomms12530 (PMC5007441; doi:10.1038/ncomms12530)
Supplement: Supplementary Information — Supplementary Figures 1-9, Supplementary Tables 1-7, Supplementary Methods and Supplementary References [file ncomms12530-s1.pdf]

Supplementary Figures

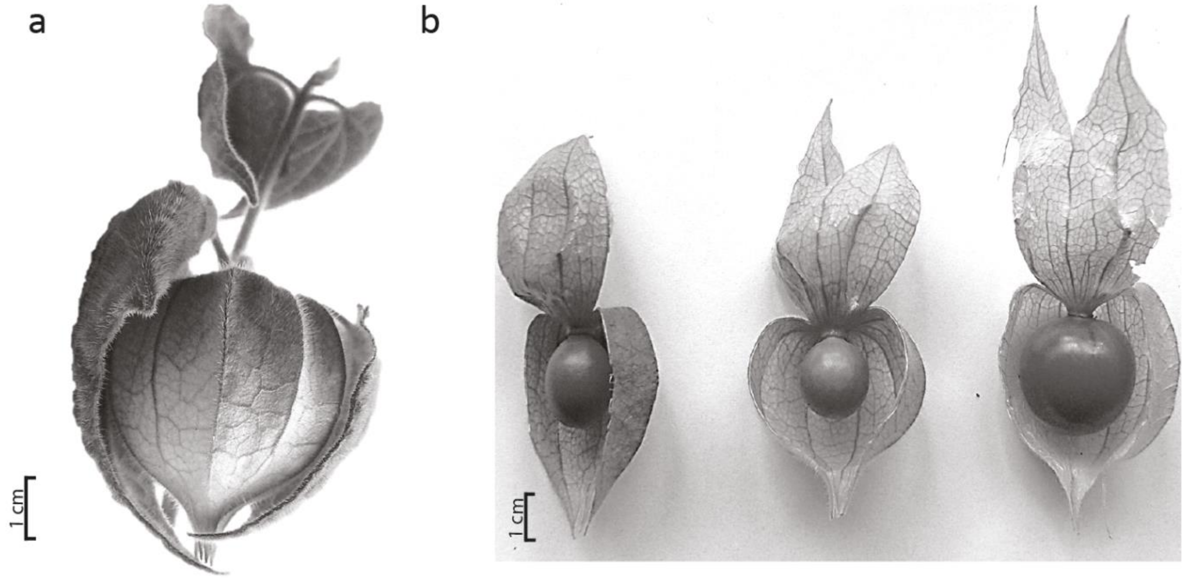

Supplementary Figure 1: ***Physalis peruviana***. (**a**) Fruit on stem and (**b**) with calyx removed. Photo © A. Barthel

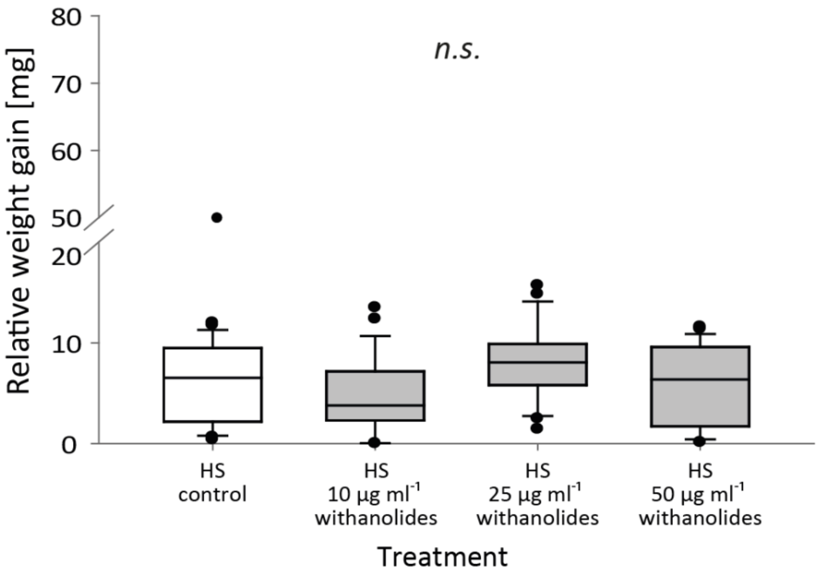

Supplementary Figure 2: Impact of withanolides on *H. subflexa* larvae. Box plot of the average relative weight gain of *H. subflexa* (HS) larvae after 7 days of exposure on diet containing 0 - 50 µg ml^{-1} withanolides. The bottom and top of the box represent the 25th and 75th percentile, respectively. The horizontal line represents median value of 24 larvae. The whiskers represent the 90th and 10th percentile and the filled circles are the extreme values (gls, *n.s.* = not significant ($P > 0.05$)).

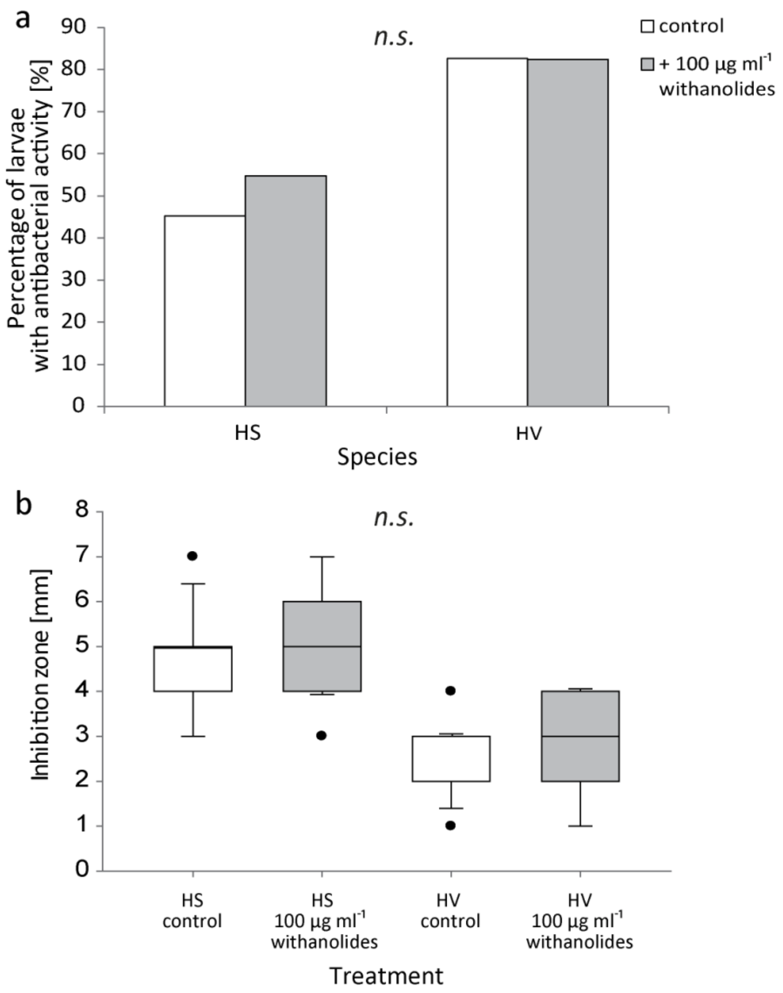

Supplementary Figure 3: Impact of withanolides on the antibacterial activity of *H. subflexa* (HS) and *H. virescens* (HV) larvae. (**a**) Proportion of larvae whose hemolymph showed a measurable antibacterial activity after 7-day exposure to 0 µg ml^{-1} (control; white) and 100 µg ml^{-1} withanolides (gray) containing diet. (**b**) Box plot of the strength of the antibacterial activity of the hemolymph of *H. subflexa* and *H. virescens* larvae that showed a measurable antibacterial activity. The bottom and top of the box represent the 25th and 75th percentile, respectively. The horizontal line represents median value of control (41 HS, 23 HV) and treated (34 HS, 17 HV) larvae. The whiskers represent the 90th and 10th percentile and the filled circles are the extreme values (glm, *n.s.* = not significant ($P > 0.05$)).

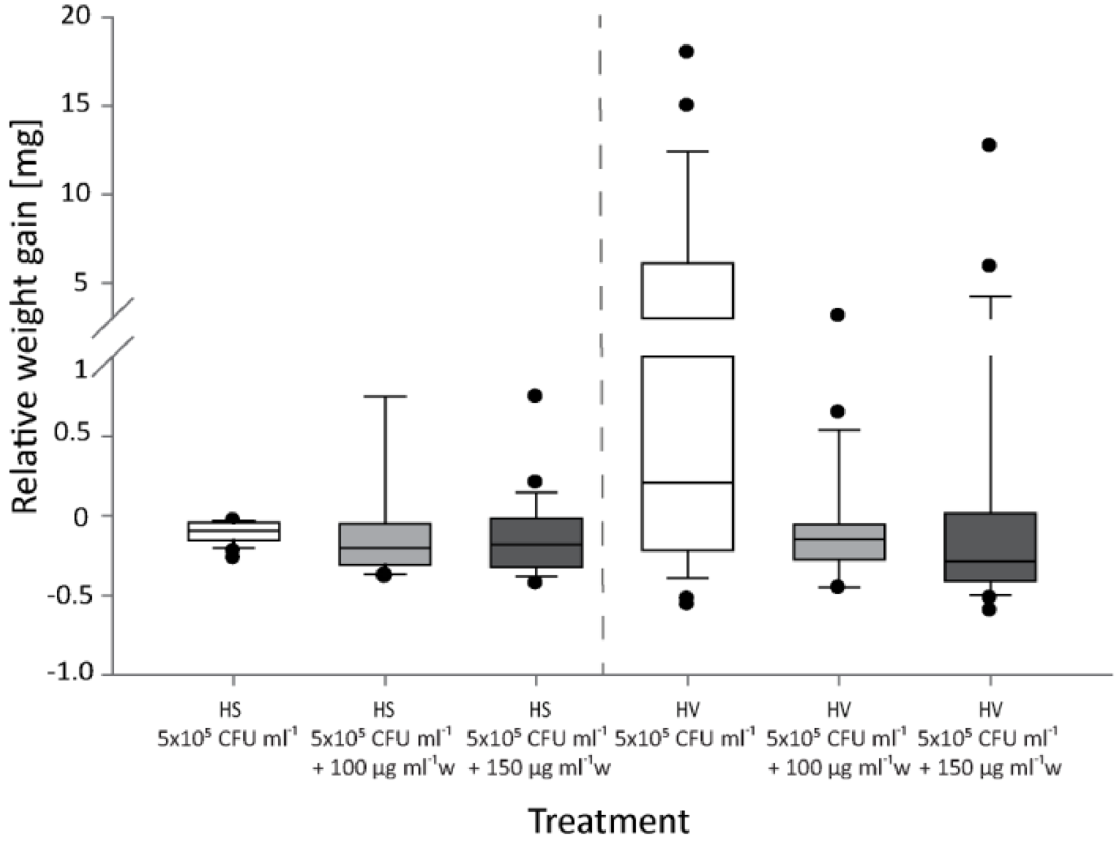

Supplementary Figure 4: High variance of the relative weight gain of infected *H. subflexa* (HS) and *H. virescens* (HV) larvae treated additionally with withanolides (w). Average relative weight gain of HS and HV larvae after 7 days of exposure to (i) $5x10^5$ CFU ml^{-1} Bt spores, (ii) $5x10^5$ CFU ml^{-1} Bt spores and 100 µg ml^{-1} withanolides and (iii) $5x10^5$ CFU ml^{-1} Bt spores and 150 µg ml^{-1} withanolides. The bottom and top of the box represent the 25th and 75th percentile, respectively. The horizontal line represents median value of control (41 HS, 23 HV) and treated (34 HS, 17 HV) larvae. The whiskers represent the 90th and 10th percentile and the filled circles are the extreme values

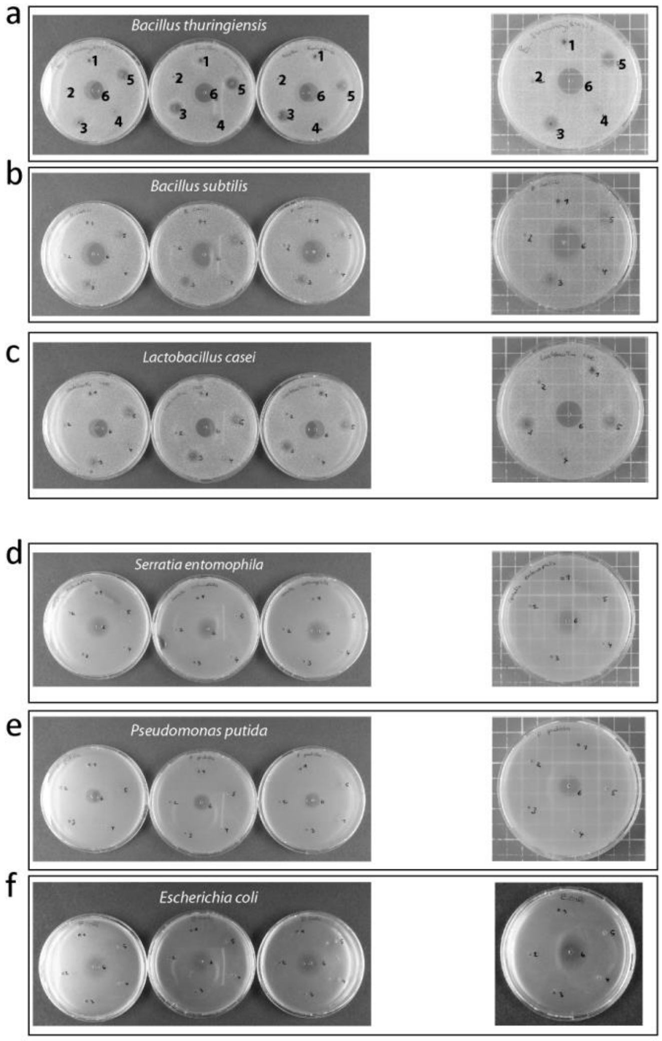

Supplementary Figure 5: Antibacterial activity of *P. peruviana* against bacterial strains. (**a**) Vegetative cells of *Bacillus thuringiensis*, (**b**) *B. subtilis* (**c**) *Lactobacillus casei* (**d**) *Serratia entomophila*, (**e**) *Pseudomonas putida* and (**f**) *Escherichia coli* were tested against their susceptibility to crude potato leaf homogenate (nightshade control) (1), crude rapeseed leaf homogenate (2), crude *P. peruviana* leaf homogenate (3), crude *P. peruviana* calyx homogenate (4), stale crude *P. peruviana* leaf homogenate and (5) 1 mg ml^{-1} gentamycin (6). Shown are triplicates and a close up of a representative plate. Numbers are shown in bold for (**a**) and the order is accordingly for the other treatments. See Supplementary Table 1 for measured inhibition zones.

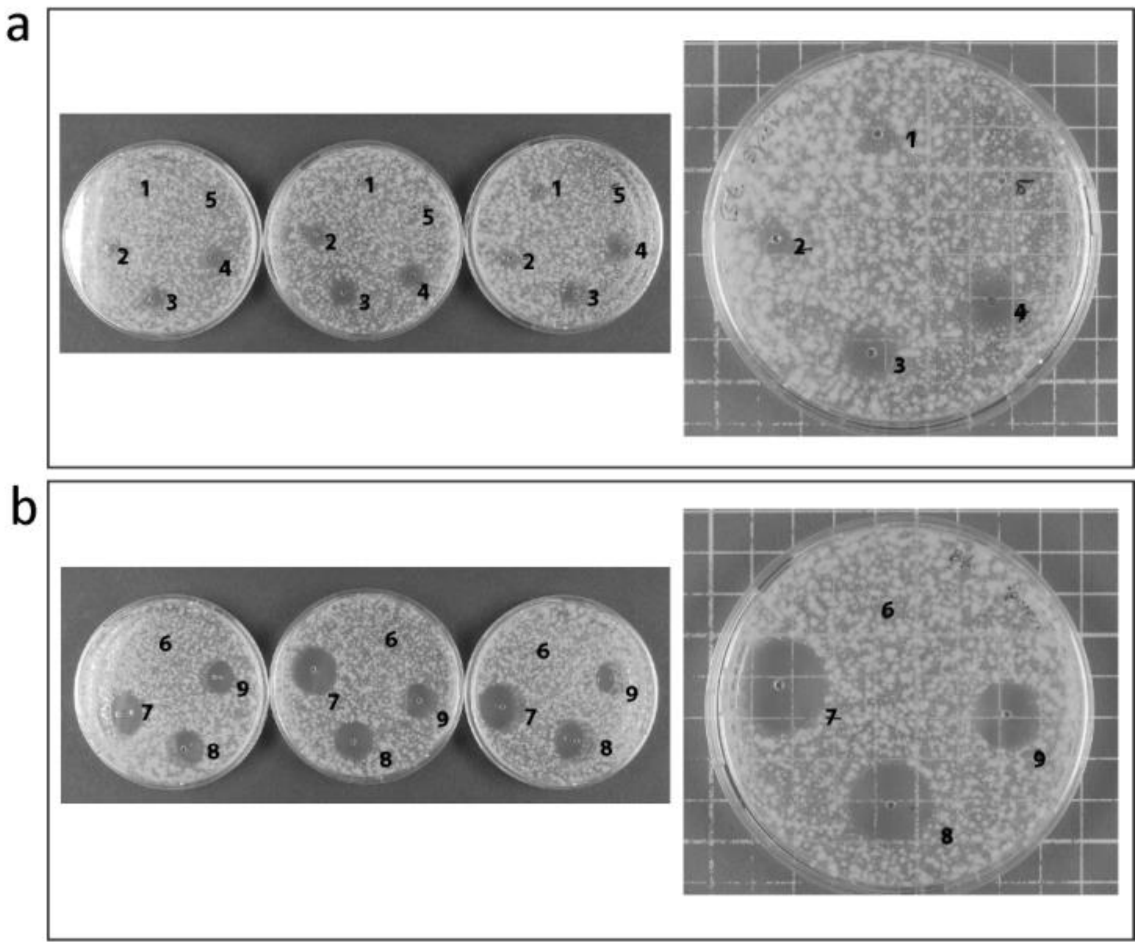

Supplementary Figure 6: Antibacterial activity of withanolides against Bt spores. Inhibition zone assay showing the antibacterial activity of (**a**) 20 µg withanolides (10 µg $µl^{-1}$) (1), 40 µg withanolides (20 µg $µl^{-1}$) (2), 60 µg withanolides (30 µg $µl^{-1}$) (3), crude *P. peruviana* leaf homogenate (4), 40 % methanol (5) and (**b**) DMSO (6), 2 µg gentamycin (1 µg $µl^{-1}$) (7), 1 µg gentamycin (0.5 µg $µl^{-1}$) (8), 0.5 µg gentamycin (0.25 µg $µl^{-1}$) (9) against Bt spores. Shown are the triplicates and a close up of a representative plate.

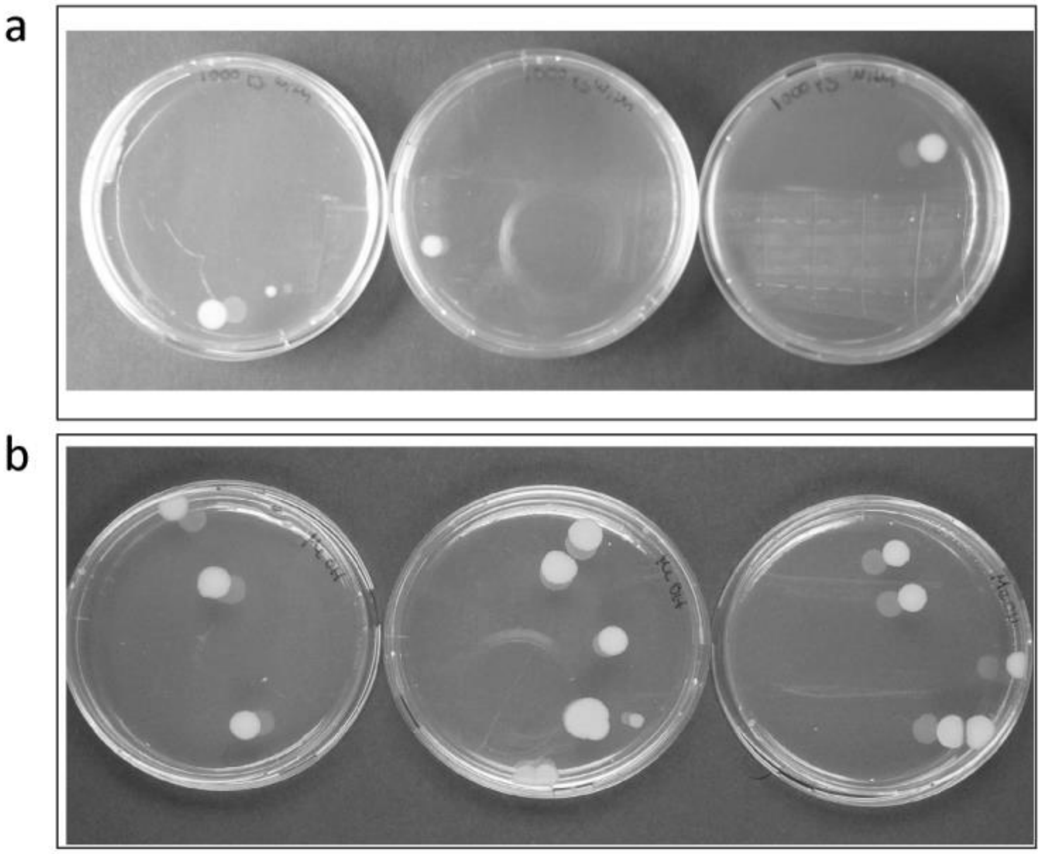

Supplementary Figure 7: Growth inhibition assay with *B. thuringiensis* spores. (**a**) *B. thuringiensis* spores grown on a LB agar (10 ml) inoculated with 100 µg ml^{-1} withanolides (solubilized in 40% Methanol). (**b**) *B. thuringiensis* spores grown on LB agar (with 40 % Methanol). Shown are triplicates.

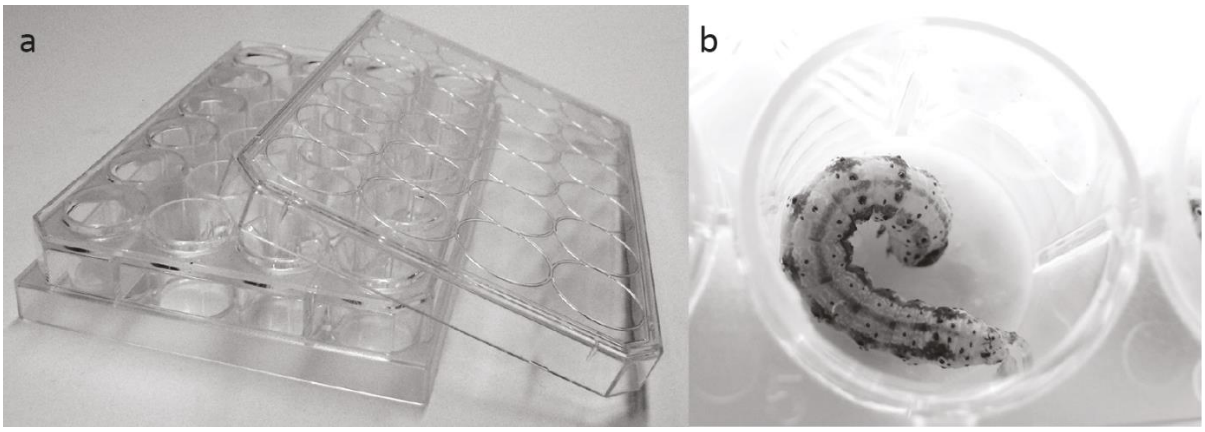

Supplementary Figure 8: Experimental setup for feeding experiments. (**a**) 24-well plates used for all feeding assays. (**b**) 4th instar larvae of *Heliothis subflexa* feeding on 1 ml artificial diet in 24-well plates.

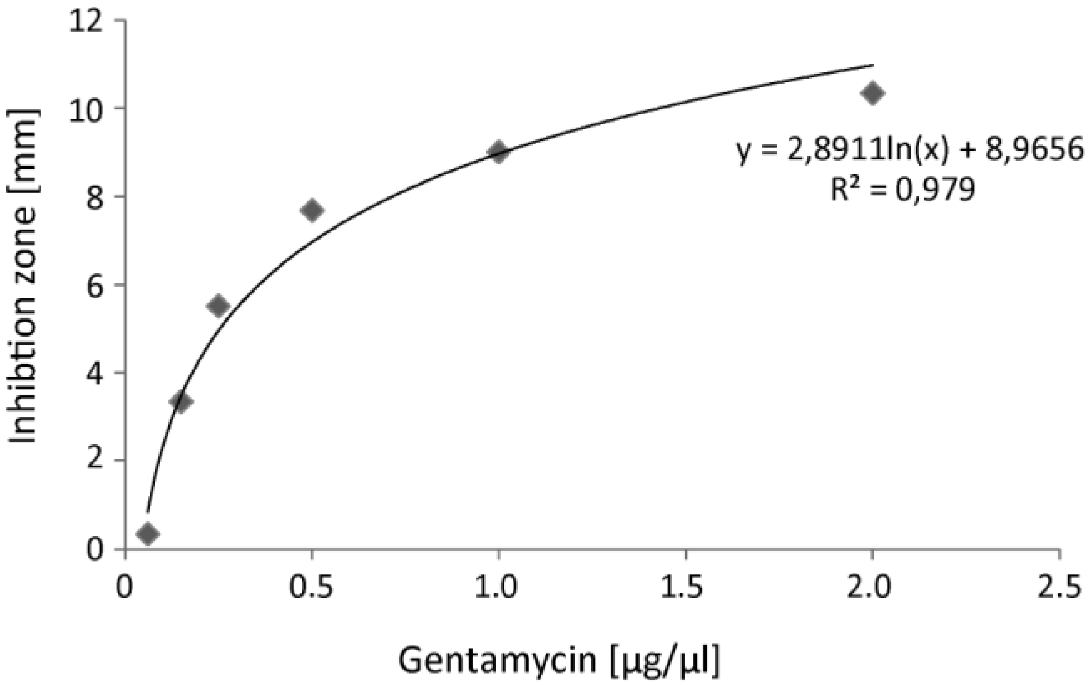

Supplementary Figure 9: Standard curve of a gentamycin dilution series. Plotted is the gentamycin concentration in µg μl^{-1} against the average inhibition zone diameter (in mm) on *B. thuringiensis* agar plates with a logarithmic trend line fitted to the data points.

Supplementary Tables

Supplementary Table 1: Measured inhibition zones of various crude leaf homogenates against different bacterial strains.

Bacteria	Treatment	Plate1 inhibition zone [cm]	Plate 2 inhibition zone [cm]	Plate3 inhibition zone [cm]
Bs	Pot	0	0,05	0
Bt	Pot	0,05	0,05	0,05
Lc	Pot	0,1	0	0,05
Se	Pot	0	0	0
Ec	Pot	0	0	0
Pp	Pot	0	0	0
Bs	Rapeseed	0	0	0
Bt	Rapeseed	0	0	0
Lc	Rapeseed	0	0	0
Se	Rapeseed	0	0	0
Ec	Rapeseed	0	0	0
Pp	Rapeseed	0	0	0
Bs	PpL	0,3	0,2	0,2
Bt	PpL	0,35	0,4	0,4
Lc	PpL	0,2	0,2	0,1
Se	PpL	0	0	0
Ec	PpL	0	0	0
Pp	PpL	0	0	0
Bs	PpC	0	0	0
Bt	PpC	0	0	0
Lc	PpC	0	0	0
Se	PpC	0	0	0
Ec	PpC	0	0	0
Pp	PpC	0	0	0
Bs	PpoL	0,1	0,1	0,1
Bt	PpoL	0,4	0,4	0,4
Lc	PpoL	0,2	0,2	0,2
Se	PpoL	0	0	0
Ec	PpoL	0	0	0
Pp	PpoL	0	0	0
Bs	Gent	1	0,9	1
Bt	Gent	0,7	0,7	0,7
Lc	Gent	0,7	0,7	0,7
Se	Gent	0,8	0,8	0,8
Ec	Gent	0,5	0,5	0,5
Pp	Gent	0,5	0,5	0,5

Abbreviations: Bs: *B. subtilis*, Bt: *B. thuringiensis*, Lc: *L. casei*, Se: *S. entomophila*, Ec: *E. coli*, Pp: *P. putida*, Pot: Crude potato leaf homogenate, Rapeseed: Crude rapeseed leaf homogenate, PpL: Crude *P. peruviana* leaf homogenate, PpC: Crude *P. peruviana* calyx homogenate, PpoL: Stale Crude *P. peruviana* leaf homogenate, Gent: gentamycin.

Supplementary Table 2: Statistic for measured inhibition zones of various crude leaf homogenates against different bacterial strains.

Comparison				F ratio	*P* <
Bacteria	Treatment	Bacteria	Treatment		
Bs	PpL	Bs	Pot	26,5	**0,000002**
Bt	PpL	Bt	Pot	193,8	**4,18E-022**
Lc	PpL	Lc	Pot	23,7	**6,3634E-06**
Bs	PpL	Bs	Rapeseed	52,4	**4,01E-010**
Bt	PpL	Bt	Rapeseed	256,2	**2,02E-025**
Lc	PpL	Lc	Rapeseed	48,4	**1,31E-009**
Bs	PpL	Bs	PpC	52,4	**4,01E-010**
Bt	PpL	Bt	PpC	256,2	**2,02E-025**
Lc	PpL	Lc	PpC	48,4	**1,31E-009**
Bs	PpoL	Bs	Pot	12,1	**0,00085566**
Bt	PpoL	Bt	Pot	213,6	**3,08E-023**
Lc	PpoL	Lc	Pot	39,2	**2,44E-008**
Bs	PpoL	Bs	Rapeseed	17,4	**0,0000821**
Bt	PpoL	Bt	Rapeseed	279	**1,79E-026**
Lc	PpoL	Lc	Rapeseed	69,8	**3,38E-012**
Bs	PpoL	Bs	PpC	17,4	**0,0000821**
Bt	PpoL	Bt	PpC	279	**1,79E-026**
Lc	PpoL	Lc	PpC	69,8	**3,38E-012**
Bs	PpL	Bs	PpoL	9,4	0,00308744
Bt	PpL	Bt	PpoL	0,5	0,4886803
Lc	PpL	Lc	PpoL	1,9	0,16821791

F ratio and *P* value of pairwise comparisons between different treatments (overall ANOVA: F ratio = 256, $P < 0.0001$ Effect tests: (bacteria: F ratio = 103, df = 5, $P < 0.0001$), (treatment: F ratio = 1507, df = 5, $P < 0.0001$), (bacteria x treatment F ratio = 36, df = 25, $P < 0.0001$)). Abbreviations: Bs: *B. subtilis*, Bt: *B. thuringiensis*, Lc: *L. casei*, Pot: Crude potato leaf homogenate, PpL: Crude *P. peruviana* leaf homogenate, PpC: Crude *P. peruviana* calyx homogenate, PpoL: Stale crude *P. peruviana* leaf homogenate.

Supplementary Table 3: Measured inhibition zones of withanolides, crude *P. peruviana* leaf homogenate, controls and gentamycin against Bt spores.

Treatment	Plate1	Plate 2	Plate3	comparison		F ratio	*P* <
	inhibition zone [cm]	inhibition zone [cm]	inhibition zone[cm]	treatment	treatment		
20w	0,1	0,2	0,2	20w	MeOH	22,5	**0,000162**
40w	0,3	0,3	0,3	40w	MeOH	72,9	**9,58E-08**
60w	0,4	0,5	0,4	60w	MeOH	152,1	**3,24E-10**
PpL	0,2	0,3	0,4	20w	DMSO	22,5	**0,000162**
MeOH	0	0	0	40w	DMSO	72,9	**9,58E-08**
DMSO	0	0	0	60w	DMSO	152,1	**3,24E-10**
Gent2	1	1	1	20w	PpL	14,4	0,001327
Gent1	0,8	0,8	0,8	40w	PpL	1E-30	1
Gent0,5	0,7	0,7	0,7	60w	PpL	14,4	0,001327

F ratios and *P* values of pairwise comparisons between withanolide treatments and control treatments (Overall t-test: F ratio = 203.4, df = 8, $P < 0.0001$). Abbreviations: 20w: 20 µg withanolides (10 µg μl^{-1}), 40w: 40 µg withanolides (20 µg μl^{-1}), 60w: 60 µg withanolides (30 µg μl^{-1}), PpL: Crude *P.peruviana* leaf homogenate, MeOH: 40% Methanol Solution, DMSO: Dimethyl sulfoxide, Gent2: 2 µg μl^{-1} gentamycin, Gent1: 1 µg μl^{-1} gentamycin, Gent0,5: 0,5 µg μl^{-1} gentamycin.

Supplementary Table 4: Growth inhibition assay with *B. thuringiensis* spores.

Treatment	
MeOH 40%	100 µg ml^{-1} withanolides
3	2
7	1
5	1

Number of colonies counted on agar plates (10 ml LB medium) inoculated with 100 µg withanolides or 40% methanol as a control (t-test, df = 1, $P < 0.038$).

Supplementary Table 5: Impact of Bt spores on the survival of *H*. subflexa.

Control vs. CFU ml^{-1} Bt spores	b	SE	Wald	df	*P*
10^3	4.201	5.802	0.524	1	0.469
10^4	2.100	2.901	0.524	1	0.469
$5x10^4$	1.433	1.037	1.911	1	0.167
$7.5x10^4$	1.127	0.558	4.070	1	**0.044**
10^5	0.909	0.420	4.691	1	**0.030**
$5x10^5$	0.870	0.297	8.608	1	**0.003**
10^6	0.708	0.260	7.417	1	**0.006**

Cox regression survival analysis of *H. subflexa* larvae treated with Bt spores. Abbreviations: b: regression coefficient of overall survival function for variable, SE: Standard error of regression coefficient, Wald: Wald statistic for variable, df: degree of freedom, *P*: Significance level for Wald statistic.

Supplementary Table 6: Impact of withanolides on the survival of *H. subflexa* (HS) and *H. virescens* (HV) larvae infected with Bt spores.

a

HS 5x10^5 CFU ml^{-1} Bt spores vs.	b	SE	Wald	df	*P*
HS 5x10^5 CFU ml^{-1} Bt spores + 100 µg ml^{-1} withanolides	-1.293	0.367	12.375	1	**0.000**
HS 5x10^5 CFU ml^{-1} Bt spores + 150 µg ml^{-1} withanolides	-0.467	0.168	7.760	1	**0.005**

b

HV 5x10^5 CFU ml^{-1} Bt spores vs.	b	SE	Wald	df	*P*
HV 5x10^5 CFU ml^{-1} Bt spores + 100 µg ml^{-1} withanolides	0.524	0.345	2.301	1	0.129
HV 5x10^5 CFU ml^{-1} Bt spores + 150 µg ml^{-1} withanolides	0.058	0.178	0.106	1	0.745

(**a**) Cox regression survival analysis of infected *H. subflexa* larvae treated with withanolides. (**b**) Cox regression survival analysis of infected *H. virescens* larvae treated with withanolides. Abbreviations: b: regression coefficient of overall survival function for variable, SE: Standard error of regression coefficient, Wald: Wald statistic for variable, df: degree of freedom, *P*: Significance level for Wald statistic.

Supplementary Table 7: Inhibition zones of withanolides against Bt.

**Treatment**	**average inhibition zone [cm]**	**standard error**	**corresponding gentamycin concentration [µg µg^{-1}]**
20w	0,167	0,033	0,048
40w	0,300	0,000	0,050
60w	0,433	0,033	0,052
PpL	0,300	0,058	0,050

Average of inhibition zones and the corresponding gentamycin concentration that would result in the same inhibition zone radius. Abbreviations: 20w: 20 µg withanolides (10 µg µl^{-1}), 40w: 40 µg withanolides (20 µg µl^{-1}), 60w: 60 µg withanolides (30 µg µl^{-1}), PpL: Crude *P. peruviana* leaf homogenate.

Supplementary Methods

Production of Bt spores

The strain HD 73 carries the Cry1Ac toxin that is toxic against various lepidopteran insects [1]. The spore solution was newly prepared beforehand using the following protocol. Bacterial cells from a glycerol stock were plated on LB agar and kept at 30 °C overnight. The following day, one bacterial colony was picked and resuspended in 5 ml LB medium and allowed to grow at 30 °C overnight on a bacterial shaker at 250 rpm. Subsequently, 100 µl of this bacterial culture was added to 50 ml HCO medium (HCO contained(l^{-1}): 7 g casein hydrolysate; 6.8 g KH_2PO_4; 0.12 g $MgSO_4$ $7H_2O$; 0.0022g $MnSO_4$ $4H_2O$; 0.014g $ZnSO_4$ $7H_2O$; 0.02 g $Fe_2(SO_4)_3$; 0.018 g $CaCl_2$ $4H_2O$; 3 g glucose; the pH was adjusted to 7.2) [2]. After seven days at 30 °C and 250 rpm, serial dilutions of this suspension were plated onto LB agar. The agar plates were incubated at 30 °C for 48 hours and germinated bacterial spores were counted (CFU ml^{-1}; colony forming units). To verify the formation of endospores, a modified spore-staining method by Wirtz-Conklin was used [3]. In short, the Bt spore suspension was coated on a glass slide and fixed with the help of a thermo plate. The fixed smear was flooded with aqueous malachite green. The slide was heated again and then counterstained with safranin. Using a light microscope, Bt spores appeared green in red-stained cells. The spore suspension was stored at 4 °C and before each application the spore concentration was newly determined.

Transcriptome sequencing, assembly and annotation

Total RNA was extracted from *H. virescens* and *H. subflexa* pooled larvae gut material with two replicates for each species per treatment, using the innuPREP RNA Mini isolation kit (Analytik Jena, Jena, Germany) according to the manufacturer's instructions. RNA integrity

was verified using an Agilent 2100 Bioanalyzer with the RNA 6000 Nano Kit (Agilent Technologies, Palo Alto, CA, USA), and RNA quantity was determined using a Nanodrop ND-1000 spectrophotometer. For each species and treatment group, 5 μg of total RNA was used for poly(A)+ mRNA purification and library preparation, and was subjected to NextGen sequencing. For each treatment two libraries were generated per species. Sequencing was carried out by the Max Planck genome Center Cologne (MPGCC) using the Illumina HiSeq-2500 platform, and utilizing the paired-end (2 x 100 bp) read technology. This yielded a total of 96 million reads for *H. virescens* and 80 million reads for *H. subflexa*. Quality control, read trimming and de novo assembly were carried out using CLC Genomics Workbench software v6.0.1 (http://www.clcbio.com) according to published methods [4]. Any conflicts among the individual bases were resolved by voting for the base with highest frequency. Contigs shorter than 250 bp were removed from the final analysis. The resulting final de novo reference assembly for *H. virescens* consisted of 37,614 contigs with a N50 contig size of 848 bp, an average contig length of 730 bp and a maximum contig length of 16,476 bp. The de novo reference assembly for *H. subflexa* consisted of 33,628 contigs with a N50 contig size of 1,329 bp, an average contig length of 896 bp and a maximum contig length of 15,361 bp.

Initial annotation of both transcriptomes through BLAST, gene ontology and InterProScan searches was performed using BLAST2GO PRO software suite v2.6.1 (www.blast2go.de) [5]. For this first round of BLAST annotations, sequences were searched against the NCBI non-redundant (nr) protein database, using an E-value cut-off of 10-3, and with predicted polypeptides of a minimum length of 15 amino acids. Second, sequences retrieving no BLASTx hit were searched again by BLASTn, against an NCBI nr nucleotide database using an E-value cut-off of 10−10. The relaxed BLAST constraints allow the identification of short putative AMPs which frequently display low E-values when searching public databases. In addition to searching against public databases, the H. virescens and H. subflexa transcriptome assemblies were also reverse BLASTed using our in-house

reference database of known AMP peptide sequences from a wide range of insect species. Subsequently, the identified candidate sequences were manually revalidated to ensure that transcripts with the same annotation were not fragmented. To derive this non-redundant set of AMPs, we manually inspected and carefully analyzed the different putative *H. virescens* and *H. subflexa* AMPs for their identity, coding sequence as well as sequence overlaps of transcripts, performing local alignments of AMP subsets belonging to the same AMP family. Using a more conservative approach we excluded from our expression analysis any protein sequences differing by a single amino acid only. However, we cannot rule out the possibility that some of the AMP sequences represent allelic variants and are not necessarily different genes because environmental heterogeneity and pathogen diversity can maintain host genetic variation in immune function.

Remapping and digital expression analysis

Digital gene expression analysis was conducted to remap the Illumina reads onto the reference transcriptomes and then by counting the sequences to estimate expression levels using QSeq Software (DNAStar Inc.). For read mapping, we used the following parameters: n-mer length = 25; read assignment quality options required at least 25 bases (the amount of mappable sequence as a criterion for inclusion) and at least 90% of bases matching (minimum similarity fraction, defining the degree of preciseness requires) within each read to be assigned to a specific contig; maximum number of hits for a read (reads matching a greater number of distinct places than this number are excluded) = 10; n-mer repeat settings were automatically determined and other settings were not changed. Biases in the sequence datasets and different transcript sizes were corrected using the RPKM algorithm (reads per kilobase of transcript per million mapped reads) to obtain correct estimates for relative expression levels. The differential expression analysis is based on replicated samples (two biological replicates

per treatment) where the geometric mean of the samples was used to calculate the respective log2 transformed RPKM values. Subsequent statistical analyses based on the log2 transformed RPKM values were performed using a moderated t-test implemented in the QSeq Software (DNAStar Inc).

Supplementary References

1 Liu, G. *et al.* Complete genome sequence of *Bacillus thuringiensis* subsp. *kurstaki* strain HD73. *Genome Announcements* **1**, e0008013, doi:10.1128/genomeA.00080-13 (2013).

2 Lecadet, M. M., Blondel, M. O. & Ribier, J. Generalized transduction in *Bacillus thuringiensis* var Berliner-1715 using bacteriophage-CP-54Ber. *J Gen Micro* **121**, 203-212 (1980).

3 Hamouda, T., Shih, A. Y. & Baker, J. R. A rapid staining technique for the detection of the initiation of germination of bacterial spores. *Lett. Appl. Microbiol.* **34**, 86-90, doi:10.1046/j.1472-765x.2002.01047.x (2002).

4 Vogel, H., Badapanda, C., Knorr, E. & Vilcinskas, A. RNA-sequencing analysis reveals abundant developmental stage-specific and immunity-related genes in the pollen beetle *Meligethes aeneus*. *Insect Mol. Biol.* **23**, 98-112, doi:10.1111/imb.12067 (2014).

5 Gotz, S. *et al.* High-throughput functional annotation and data mining with the Blast2GO suite. *Nucleic Acids Res.* **36**, 3420-3435, doi:10.1093/nar/gkn176 (2008).
